# Neurobehavioral alternations of the female offspring born to polycystic ovary syndrome model rats administered by Chinese herbal medicine

**DOI:** 10.1186/s13020-021-00512-4

**Published:** 2021-10-02

**Authors:** Xian Zhang, Lifang You, Xiaohui Zhang, Fangfang Wang, Yi Wang, Jue Zhou, Chang Liu, Fan Qu

**Affiliations:** 1 Women’s Hospital, School of Medicine, Zhejiang University, 1 Xueshi Road, Hangzhou, 310006 China; 2 First People’s Hospital of Yuhang District, Hangzhou, 311103 Zhejiang China; 3 Pharmaceutical Informatics Institute, College of Pharmaceutical Sciences, Zhejiang University, Hangzhou, 310058 China; 4 College of Food Science and Biotechnology, Zhejiang Gongshang University, Hangzhou, 310018 China; 5 The Second Clinical Medical College of Zhejiang, Chinese Medical University, Hangzhou, 310053 China

**Keywords:** Molecular network, Liquid chromatography coupled with mass spectrometry (LC–MS), Network pharmacology, Polycystic ovarian syndrome (PCOS), Bu-Shen-Tian-Jing formula (BSTJF), Neurobehavioral manifestations

## Abstract

**Background:**

Chinese herbal medicine (CHM) has significant effects that improve the reproductive functions of patients with polycystic ovary syndrome (PCOS). However, the intergenerational effects of CHM on offspring and the underlying mechanism of CHM remain unclear. This study aimed to explore the effects and the underlying mechanism of CHM, specifically the Bu-Shen-Tian-Jing formula (BSTJF), on model rats with polycystic ovary syndrome (PCOS) and the neurobehavioral alterations of female offspring born to PCOS rats administered BSTJF.

**Methods:**

High-performance liquid chromatography-mass spectrometry (HPLC–MS) and network pharmacology analysis were performed to identify the active ingredients and potential targets of BSTJF. Moreover, PCOS model rats were used to validate the role of BSTJF in reproduction and progeny neural development and to confirm the network pharmacological targets.

**Results:**

A total of 91 constituents were characterized from BSTJF. The 20 most significant KEGG pathways and the high-frequency genes of these pathways were predicted to be putative targets of these molecules. The rat experiment showed that the downregulation of FOS protein expression in the ovarian granulosa cells of the PCOS group was reversed by BSTJF. The target residence time of the 5-week-old female offspring of the BSTJF group was higher than that of the PCOS group in the water maze experiment. Compared to the PCOS group, the changes in dendritic spine density, ultrastructure of neurons and synapses, and Gabrb1 and Grin2b protein expression levels in the hippocampus of female offspring were partially reversed in the BSTJF group.

**Conclusions:**

BSTJF can effectively improve ovarian follicle development in PCOS rats and has positive effects on pubertal neurobehavioral alterations in the female offspring of these rats by reversing dendritic spine density, the ultrastructure of neurons and synapses, and the Gabrb1 and Grin2b protein expression levels in the hippocampus.

**Supplementary Information:**

The online version contains supplementary material available at 10.1186/s13020-021-00512-4.

## Background

Polycystic ovary syndrome (PCOS) is a prevalent gynecological endocrine disease affecting the health of reproductive-aged women [1]. The disease cannot be completely treated by a single treatment or measure due to its heterogeneity [2, 3]; therefore, the development of successful therapeutic strategies remains a clinical challenge.

Recently, traditional Chinese medicine (TCM) has gained increasing acceptance worldwide for its long-term clinical application in various gynecological diseases, and multiple studies have indicated the efficacy of Chinese herbal medicine [4]. Recent studies have demonstrated that herbal formulas or compounds isolated from herbs have beneficial effects in PCOS patients and animal models [5–11].

According to the clinical manifestations, PCOS can be attributed to "amenorrhea," "infertility," "masses in the abdomen," or "obesity" in TCM [12]. In the past 30 years, TCM research data have illustrated that PCOS is mainly caused by a dysfunctional relationship among the kidney, Chong Ren, and uterus [13]. The “Kidney-Tian Gui-Chong Ren-uterus” theory of TCM resembles the “hypothalamic-pituitary axis” theory in modern medicine [14]. Kidney deficiency is the main pathological mechanism of ovulatory dysfunctional infertility [12, 14, 15]. Therefore, in TCM, PCOS should be treated mainly by nourishing the kidney [12, 15].

However, TCM formulas are complex mixtures containing hundreds of different ingredients [16], which mainly work through multicomponent, multitarget, and multilevel methods [17]. The exact mechanism of TCM in PCOS remains unclear. High-performance liquid chromatography (HPLC) coupled with mass spectrometry (MS) has been the preferred method for TCM research because of its excellent resolution, accuracy, and sensitivity [18–20]. Because LC–MS analyses may provide several data sets, making it challenging to deal with detected chemical signatures [21], molecular networks are used to aid the visualization and interpretation of the compounds detected in an MS experiment and the chemical relationships between them. Although the wider application of molecular networking has been recognized since 2012 [22], this approach has been successfully applied in drug discovery, drug metabolism studies, and the medical field [23]. Based on the rapid progress in systems biology, network pharmacology is a powerful approach for illustrating and analyzing complexities among drug combinations from a systematic perspective [24]. Network pharmacology has been used in studies to detect effective substances and determine the TCM mechanisms against many diseases [25].

To explore the effects of TCM on PCOS and the underlying mechanism, the Bu-Shen-Tian-Jing formula (BSTJF), a clinically supported formula in tonifying the kidney, was examined as a representative prescription in this study. BSTJF is a patented Chinese herbal compound owned by the corresponding author’s team (International patent PCT: Publication No. WO2012/100471, Application No. PCT/CN2011/073938), which has been clinically shown to significantly improve pregnancy outcomes and alleviate hyperandrogenism in PCOS rats induced by testosterone propionate [26].

In our previous study, FOS protein levels were found to be downregulated in the placental villi of the PCOS group [27]. We also found that Gabrb1, Grin2b, and Adra1b, which are enriched in the neuroactive ligand-receptor interaction pathway, were expressed differently (compared with the controls) in the hippocampus of female offspring of PCOS model rats by analyzing transcriptomic data and KEGG pathway enrichment [27]. Many studies have found that PCOS affects the neural development of progeny, such as autism spectrum disorder [28], attention deficit hyperactivity disorder [29], anxiety and emotional borderline symptoms [30]. Although TCM has a positive effect on the neural development of offspring [31, 32], there are few studies on the effect of TCM, particularly the TCM formula, on the nervous system of PCOS offspring.

In the present work, LC–MS analysis was employed to determine the active constituents of BSTJF, and a molecular network was applied to profile the chemical composition of the formula. Network pharmacology was then performed to investigate the correlations among the constituents, potential protein targets, and related signaling pathways. Moreover, a study on PCOS model rats was conducted to evaluate the effects of BSTJF on PCOS, and double-labeling immunofluorescence was used to validate the potential targets. Furthermore, neurobehavioral alterations of the female offspring born to the PCOS rats administered with BSTJF were observed, and the underlying mechanism was explored by immunohistochemistry and western blotting.

## Methods

### Materials and reagents

BSTJF consists of seven herbs, including H. *glutinosa* Libosch. (Dihuang), 20 g; *Ligustrum lucidum* Ait. (Nvzhenzi), 20 g; *Rubus chingii* Hu (Fupenzi), 10 g; *Cwscwia australis* R. Br. (Tusizi), 15 g; *Psoralea corylifolia* L. (Buguzhi), 15 g; *Astragalus membranaceus* (Fisch.); *Bge. var. mongholicus* (Bge.); Hsiao (Huangqi), 10 g; and *Salvia m iltiorrhiza Bge.* (Danshen), 10 g, which were purchased from Huadong Medicine Co., Ltd. The reference standards, including catalpol, sodium danshensu, 3,4-dihydroxybenzaldehyde, neochlorogenic acid, salidroside, chlorogenic acid, *p*-coumaric acid, calycosin-7-*O*-beta-d-glucoside, rutin, rosmarinic acid, salvianolic acid B, and salvianolic acid A, were purchased from Shanghai Winherb Medical Technology Co., Ltd. (Shanghai, China). Adenosine, rehmannioside D, nuezhenidic acid, psoralenoside, isopsoralenoside, hyperoside, acteoside, salvianolic acid D, specneuzhenide, ononin, salvianolic acid E, quercetin, calycosin, and isopsoralen were provided by Shanghai Yuanye Bio-Technology Co., Ltd. (Shanghai, China). Schaftoside and astragalin were obtained from Chengdu Must Biotechnology Co., Ltd. (Chengdu, China). Sucrose was purchased from Aladdin Industrial Corporation (Shanghai, China). Psoralen was purchased from the National Institutes for Food and Drug Control (Beijing, China). HPLC-grade acetonitrile, methanol, and formic acid were obtained from Merck (Darmstadt, Germany). The samples were stored at 4 °C and the reference standards were stored at − 20 °C at the Pharmaceutical Informatics Institute of Zhejiang University. Deionized water was prepared using an Elga PURELAB flex system (ELGA LabWater, UK). All other chemicals and reagents used were of analytical grade.

Testosterone propionate was purchased from Beijing Solaibao Technology Co., Ltd. (Beijing, China). Sesame oil (S3547) was purchased from Sigma–Aldrich (Steinheim, Germany). Pregnant mare serum (PMSG) and human chorionic gonadotropin (HCG) were obtained from Ningbo Second Hormone Factory (Ningbo, China). Sump dye was purchased from Haoke Biotechnology Co., Ltd. (Hangzhou, China). Primary antibodies against Foxl2, ERK1/2, Grin2b, and Adra1b were procured from Proteintech Group, Inc. (Rosemont, USA). Primary antibodies against c-FOS and Gabrb1 were obtained from Affinity Biosciences, Ltd. (Jiangsu, China). The secondary antibody goat anti-rabbit IgG H&L (HRP) was provided by Abcam (Cambridge, UK). TSA-FITC and TSA-CY3 were purchased from Pinuofei Biotechnology Co., Ltd. (Wuhan, China). Ziehl and Golgi dye kits were obtained from ServiceBio (Wuhan, China).

### Pharmacological research on BSTJF

#### LC–MS analysis of BSTJF

BSTJF was prepared by soaking all the raw materials in water for 30 min and then decocting it for 60 min. Standard stock solutions of 30 chemical compounds were prepared at a concentration of 1 mg/ml by dissolving them in 70% methanol solutions and then mixing. All samples were centrifuged at 10,000 rpm for 20 min. The supernatants were collected and subjected to LC–MS analysis.

An Acquity UPLC system (Waters, Milford, MA, USA) coupled with a Triple TOF 5600plus MS (AB SCIEX, Framingham, MA, USA) was employed for chemical identification. Chromatographic separation was performed on a Waters ACQUITY UPLC HSS T3 column (100 mm × 2.1 mm i.d. 1.8 μm) at 35 °C with mobile phase A (0.1% formic acid–water) and mobile phase B (0.1% formic acid-40% acetonitrile–water). The flow rate was 0.25 ml/min and a linear gradient elution was programmed: 0–7 min, 0–5%B; 7–14 min, 5–12%B; 14–25 min, 12–16%B; 25–40 min, 16–25%B; 40–50 min, 25–35%B; 50–60 min, 35–55%B; 60–62 min, 55–65%B; 62–65 min, 65–75%B; 65–68 min, 75–100%B; 68–73 min, 100–100%B. The injection volume was 3μL. Mass spectrometry analysis was performed in positive and negative mode with the following parameters: scan range, m/z 90–1500; ion source GS1, 50 psi; ion source GS2, 50 psi; curation gas (CUR), 35 psi; temperature, 600 °C for ESI^+^ and 550 °C for ESI-; ion spray voltage (IS), -4.5 kV for ESI-; and 5.0 kV for ESI^+^.

#### Molecular networking and network pharmacology analyses

The molecular network was constructed as previously reported [33]. MS Convert and MZ mine 2.53 were used for LC–MS/MS data processing.

Mass detection was performed by fixing the noise level at 100 for MS1 and 0 for MS2. Chromatogram building was achieved using the ADAP chromatogram builder with a minimum group size of 5 scans, a group intensity threshold of 100, a minimum highest intensity of 100 and a m/z tolerance of 0.01 Da (or 10 ppm). For chromatogram deconvolution, the wavelet (ADAP) method was selected with the following settings: S/N threshold = 10, minimum feature height = 100, coefficient/area threshold = 50, peak duration range 0.00–10.00 min, and retention time (RT) wavelet range 0.0–0.50. MS/MS scans were paired using a m/z tolerance range of 0.02 Da and RT tolerance range of 1 min. The isotopic peak grouper algorithm was used with a m/z tolerance of 0.01 Da (or 10 ppm) and an RT tolerance of 0.1 min. Thereafter, the peak list was filtered using a peak list row filter to keep only peaks with the MS2 scan and reset the peak number ID. Finally, the data file, including the retention time, peak area, and other information of the compounds, were exported.

The corresponding molecular networking was created according to the online workflow at GNPS (http://gnps.ucsd.edu) [34]. In this analysis, the parent mass tolerance and fragment ion mass tolerance were set at 0.02 Da. The minimum cluster size was set at 1, the network topology was 20, and the maximum connected component size was 200. In addition, the MS cluster and filter precursor window tools were turned off. The network was created with a cosine score above 0.7 and more than six matched peaks. The molecular networking data were visualized using Cytoscape (version 3.8.0).

The ingredients identified by UPLC-Q/TOF–MS analysis of BSTJF were considered candidate compounds. Symmap database (https://www.symmap.org/) [35], BATMAN database (http://bionet.ncpsb.org/) [36] and TargetNet database (http://targetnet.scbdd.com) [37] was used to predict the putative targets. The selection criteria of the target were as follows: for the Target-Net database, targets with area under the receiver operating characteristic curve ≥ 0.7 and probability > 0.9 and targets with score cutoff > 20 were retained. Then, all targets were converted to official gene symbols using the UniProt database (https://www.UniProt.org/). KEGG pathway enrichment analysis was performed using the DAVID database (https://david.ncifcrf.gov/) [38].

### The effects of BSTJF on the PCOS model rats

#### Establishment of the PCOS rat model

All animal studies were approved by the Ethical Review Committee of the Experimental Animal Welfare, Zhejiang University (Zhejiang, China), according to the European Community guidelines (EEC Directive of 1986; 86/609/EEC). Forty-five neonatal female Sprague–Dawley rats, provided by Vital River (Beijing, China), were randomly divided into the PCOS model and control groups (n = 30 in the PCOS group and n = 15 in the control group). All animals were fed a standard laboratory diet and housed under a 12:12 h light/dark cycle at 25 °C.

Nine days after birth, the PCOS group was induced by subcutaneous injection of testosterone propionate at a dose of 0.1 mg/0.004 ml sesame oil per g of animal [39], and the controls only received sesame oil. When the rats were 8 weeks old, the estrous cycle of the rats was measured for 14 consecutive days by analyzing the vaginal smears cell types [40]. For the PCOS group, we retained only rats with irregular vaginal smears for the subsequent experiments. In addition, two rats in each group were randomly sacrificed to obtain ovarian tissue for model verification. Then, the PCOS model rats were randomly divided into the PCOS and BSTJF groups.

#### Intervention with BSTJF on PCOS rat

The BSTJF decoction was as follows: A total of 100 g of raw herb (the composition of BSTJF is described in 2.1. of the Materials and reagents section), a daily dose for adults, were decocted with water 10 times for each decoction with three total decoctions (1 h each). The decoction solutions were then combined, filtered, and concentrated into a solution with a concentration of 2.00 g/ml, which was hermetically stored at 4 °C.

The BSTJF group received a BSTJF decoction infusion once a day at a dose of 333 mg/200 g for 4 consecutive weeks, whereas the control and PCOS groups only received the same volume of distilled water. The estrous cycle was observed during the last 2 weeks of infusion, and the ovaries were removed from the sacrificed rats (4 rats in each group) and fixed in 4% paraformaldehyde for morphological observation by hematoxylin and eosin (H&E) staining.

#### Double-labeling immunofluorescence

Double-labeling immunofluorescence was used to detect the expression of target proteins in ovarian granulosa cells according to the manufacturer's instructions. Ovarian granulosa cells were specifically labeled with anti-Foxl2 antibody [41], and the target proteins were labeled with the corresponding antibodies.

Two slides were prepared for double-labeling immunofluorescence, and the samples on the slides were cut from each ovary sample. The slides were blocked with 3% bovine serum albumin (BSA) for 30 min at room temperature (RT) (20–25 °C). The slides were then incubated with the primary antibodies overnight at 4 °C. The following antibodies were used: anti-ERK1/2 (1:200; Proteintech Group, Inc., Rosemont, USA; no. 11257–1-AP) and anti-FOS (1:200; Affinity Biosciences, Ltd., Jiangsu, China; no. AF0132). The slides were then incubated with goat anti-rabbit IgG H&L (HRP) (1:2000; Abcam, Cambridge, UK; no. Ab205718) secondary antibody for 50 min at RT. Then, anti-Foxl2 (1:200; Proteintech Group, Inc., Rosemont, USA; no. 19672–1-AP) was added to the slide and incubated overnight at 4 °C. Thereafter, goat anti-rabbit IgG H&L (HRP) (1:2000; Abcam, Cambridge, UK; no. Ab205718) secondary antibody was incubated with the sample for 50 min at RT. Nuclei were stained with 40,6-diamidino-2-phenylindole (DAPI). Fluorescence signals were visualized using a confocal laser microscope. The value of Foxl2 and target protein double-positive cell number/FoxL2 positive cell number were used to describe the expression rate of ovarian granulosa cells to the target protein.

### Observation of the neurobehavioral manifestations and morphology of the dentate gyrus of female offspring born to PCOS rats administered by BSTJF

#### Offspring acquisition

The three groups of rats were injected intraperitoneally with 20 IU pregnant mare serum (PMSG) at 5:00 pm, followed by 20 IU of human chorionic gonadotropin (HCG) after 48 h [42] and were immediately mated with male rats at a scale of 1:1. Once vaginal sperm plugs were observed the following day, the female rats were raised separately, and their weights were monitored twice a week to determine if they were pregnant. The dams were allowed to deliver the pups naturally, and the birth date of progeny was considered postnatal day (PND) 0. The pups were weaned on PND 21, and then the female and male pups were fed separately.

#### Learning and memory testing with the Morris water maze in the female offspring

The Morris water maze test was performed on female offspring at 5 weeks (puberty) and 13 weeks (adulthood) old. Three progeny females were randomly selected from the progeny of each female parent to participate in the experiment. The water maze apparatus consisted of a circular water tank 1.5 m in diameter and was filled to a depth of 60 cm with water (22 ± 2 °C). The tank was divided into four quadrants. A circular platform was placed in one of the quadrants (target quadrant), 1 cm below the water surface. The rats were subjected to three learning sessions per day for 5 days and a memory retention test (probe test) on the fifth day. Subsequently, an estrous cycle was observed after the probe testing. In the learning sessions, the rats were placed at the same starting positions per quadrant and allowed to freely swim and find the hidden platform (target) for 60 s. The time it took a rat to reach the platform after it was placed in the water was recorded as the latency to the target. If the rat did not locate the platform within 60 s, it was guided to the platform and remained there for 10 s, and its latency to target was recorded as 60 s. On the fifth day of the probe test, the platform was removed, and the rats were allowed to swim freely for 60 s. One female offspring rat at the diestrus stage was randomly taken from each family. The distance to the target, latency to the target in the training sessions, and the distance, time, entries in the target zone and in the target quadrant during the probe test were recorded using a camera linked to a computerized video tracking system (DMS-2MORRIS water maze system, Institute of Medicine, Chinese Academy of Medical Sciences. Beijing, China).

#### Brain tissue collection of the female offspring rats

After completing the Morris water maze test, the female offspring were anesthetized with sodium pentobarbital and transcardially perfused with 100 ml of saline followed by 200 ml of a fixative containing 2% glutaraldehyde and 2% paraformaldehyde in 0.01 mol/l phosphate buffer (PB, pH 7.4). Then, the brains were removed from the skulls and cut sagittally in half. The left hemispheres were stored in a fixative solution at 4 °C for subsequent paraffin embedding. For the right hemispheres, the dentate gyrus of the hippocampus in some of the samples from each group was exfoliated completely and cut into 1 × 3 mm pieces to be preserved in glutaraldehyde for subsequent electron microscope observation. The remaining samples of each group were fixed for Golgi staining or the hippocampus was removed and stored at − 80 °C.

#### Nissl staining

The left hemisphere samples were paraffin-embedded and sectioned coronally into 4-μm slices in the dentate gyrus area. Nissl staining was used to observe the morphology of the dentate gyrus of female offspring. The results were examined under a light microscope. (DM2500, Leica Microsystems, Germany).

#### Golgi staining

Golgi staining was used to observe the morphology of dendrites and analyze the density of dendritic spines in the dentate gyrus. The hemisphere tissue of the female offspring was stained with Golgi dye according to the manufacturer's instructions and then sliced. Image J software was used to analyze the Golgi-stained images, and dendritic spines were observed under a 400-fold microscope. Starting from the first branch of the dendritic cells from the cell body, the number of dendritic spines in the 30–60 μm length range was calculated, and the density was obtained.

#### Transmission electron microscopy

Transmission electron microscopy was used to observe the ultrastructure of neurons and synapses in the dentate gyrus of female offspring. First, the dentate gyrus tissue was cut into 1 × 3 mm blocks and stored in glutaraldehyde for 2 h at room temperature. Thereafter, the samples were rinsed with BP buffer, postfixed with 2% osmium tetroxide for 1–2 h, rinsed again with BP buffer, dehydrated in an ethanol series, infiltrated in a mixture of Spurr and acetone, embedded in 100% Spurr embedding agent overnight and polymerized for 24 h, and then the samples were cut into 70 nm thick slices for transmission electron microscopy (Hitachi H-7650, Japan) observation using a side-mounted CCD camera (Model 830 SC200, Gatan, USA) at 8000×, 25000×, and 50,000× magnification.

#### Immunohistochemistry

To further investigate the mechanism of action of BSTJF on female offspring born to PCOS, based on our previous work [27], immunohistochemistry was used to observe the expression of Gabrb1, Grin2b, and Adra1b in the dentate gyrus of female offspring rats. Anti-Gabrb1 (1:100; Affinity Biosciences, LTD., Jiangsu, China; no. AF6207), anti-Grin2b (1:50; Proteintech Group, Inc., Rosemont, USA; no. 21920–1-AP), and anti-Adra1b (1:100; Proteintech Group, Inc., Rosemont, USA; no. 22419–1-AP) were used as primary antibodies at RT for 1 h, followed by incubation with hypersensitive rabbit/mouse universal secondary antibody (Tuling Hangzhou Biomedical Co., Ltd; Hangzhou, China; no. I20012C) for 30 min at RT. Digital images of the dentate gyrus were captured using a light microscope (ECLIPSE E100, NIKON, Japan). Gabrb1-, Grin2b-, and Adra1b-immunoreactive neurons were counted, and the positive rate was described by integrated optical density (IOD).

#### Western blotting

The hippocampal tissue was fully ground with a mortar and pestle, followed by treatment with lysate. Next, ultrasound was used to turn the tissue into a homogenate, which was centrifuged at 12,000 rpm for 10 min at 4 °C, and the supernatants were collected. The protein concentration was measured by bicinchoninic acid (BCA) assay. Protein was subjected to sodium dodecyl sulfate polyacrylamide gel electrophoresis (SDS–PAGE 8%) and transferred to polyvinylidene difluoride membranes (soaked in methanol approximately 10 s before use) in transfer buffer. The membranes were incubated for 2 h in blocking solution (5% skim milk). Then, the membranes were washed with TBST and incubated with primary antibodies at 4 °C overnight. The membranes were washed three times in TBST for 10 min each time and incubated with secondary antibody for 1 h at room temperature. β-actin was used as a loading control. The immune complexes were detected using enhanced chemiluminescence. The density of specific bands was analyzed using Image J software.

The dilution ratios of primary antibodies were as follows: anti-Gabrb1 (1:1000; Affinity Biosciences, LTD., Jiangsu, China; no. AF6207); anti-Grin2b (1:1000; Proteintech Group, Inc., Rosemont, USA; no. 21920–1-AP); and anti-Adra1b (1:1000; Proteintech Group, Inc., Rosemont, USA; no. 22419–1-AP).

### Statistics analysis

Data are presented as the mean ± SD. Statistical significance was determined using one-way ANOVA or non-parametric test for multiple comparisons and LSD test for pairwise comparisons between groups. Fisher’s exact test was used for comparisons among categorical variables. Statistical significance was set at p < 0.05.

## Results

### Results for the pharmacological study

#### Characterization of the chemical constituents in BSTJF

**Fig. 1:**
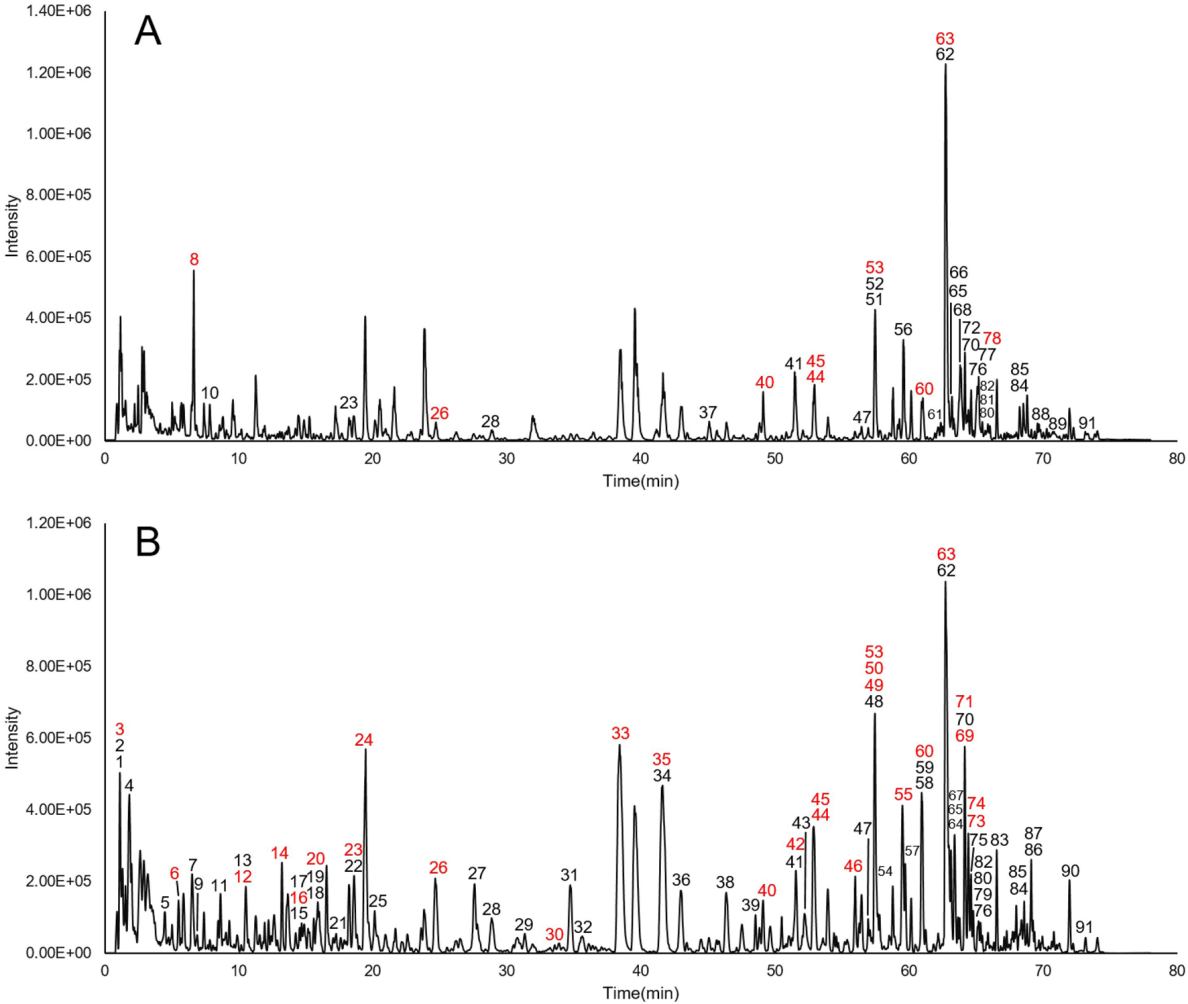
Base peak chromatograms of BSTJF. **A** Positive mode. **B** Negative mode. Red numbers represent compounds that were identified with reference standards

The representative UPLC-Q-TOF/MS chromatograms of BSTJF are shown in Fig. 1A, B. A total of 91 constituents were characterized from BSTJF based on literature and database matching. The compounds identified from BSTJF are listed in the Additional file 1, including 20 glycosides, 19 flavonoids, 19 phenylpropyl acids, 4 saccharides, 3 coumarins, 2 alkaloids, 1 steroid, and other types of compounds. Among them, 30 compounds were identified by comparison with reference standards in regard to the retention time and mass spectra.

**Fig. 2:**
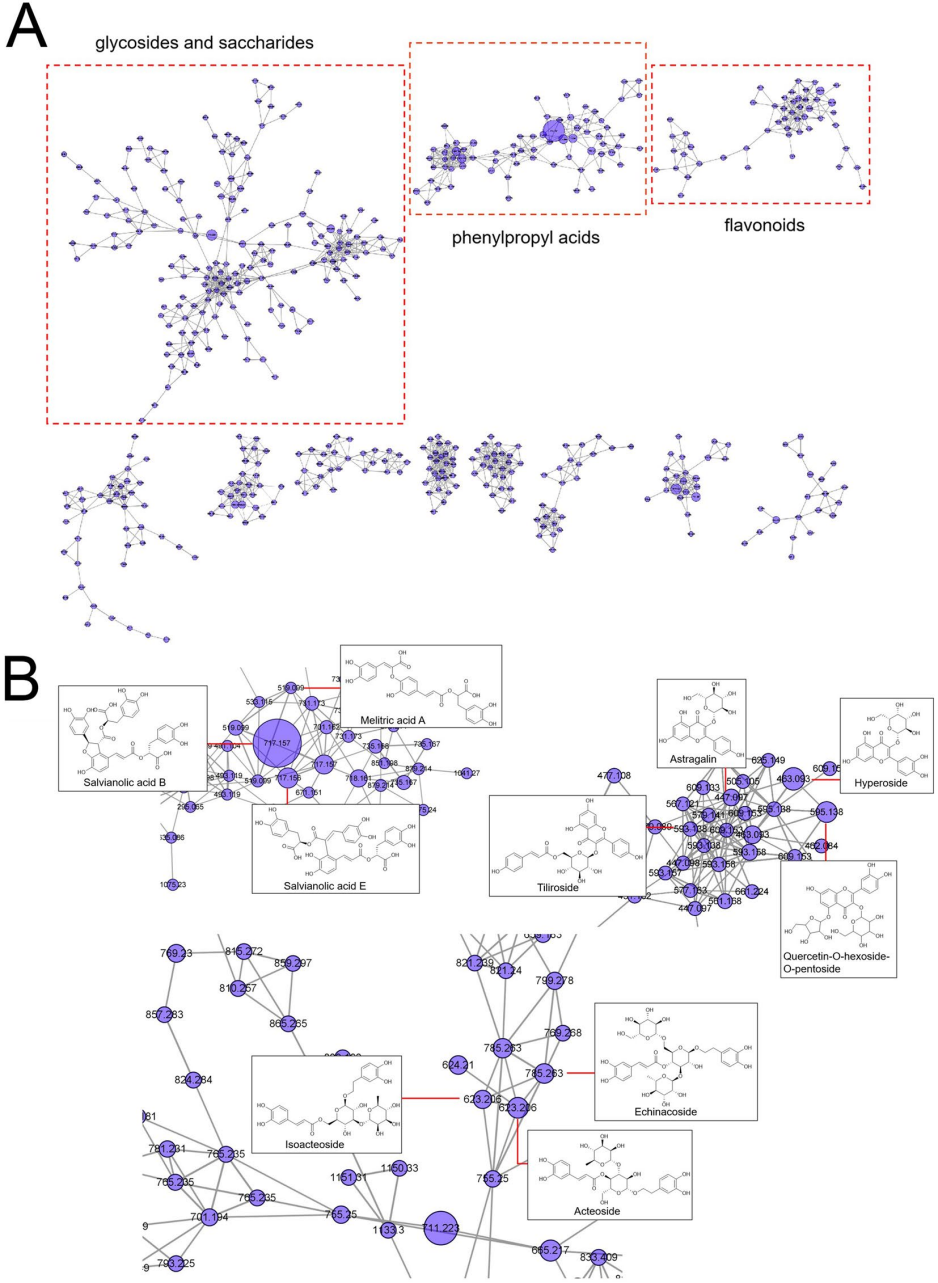
Molecular networking of BSTJF. **A** Main clusters of molecular networking. **B** Representative structures of main clusters

The molecular network of BSTJF was used to visualize the structural relationships in the samples. In the molecular network, the size of the nodes represents the peak area of each compound in the sample (Fig. 2A, B). Three major clusters were highlighted in the molecular network, which represented glycosides, saccharides, phenylpropyl acids, and flavonoids. The representative compounds were positioned and annotated in the network. Glycosides and saccharides aggregate to form the largest cluster, while salvianolic acid B has the highest peak area.

#### Network pharmacology analysis

**Fig. 3:**
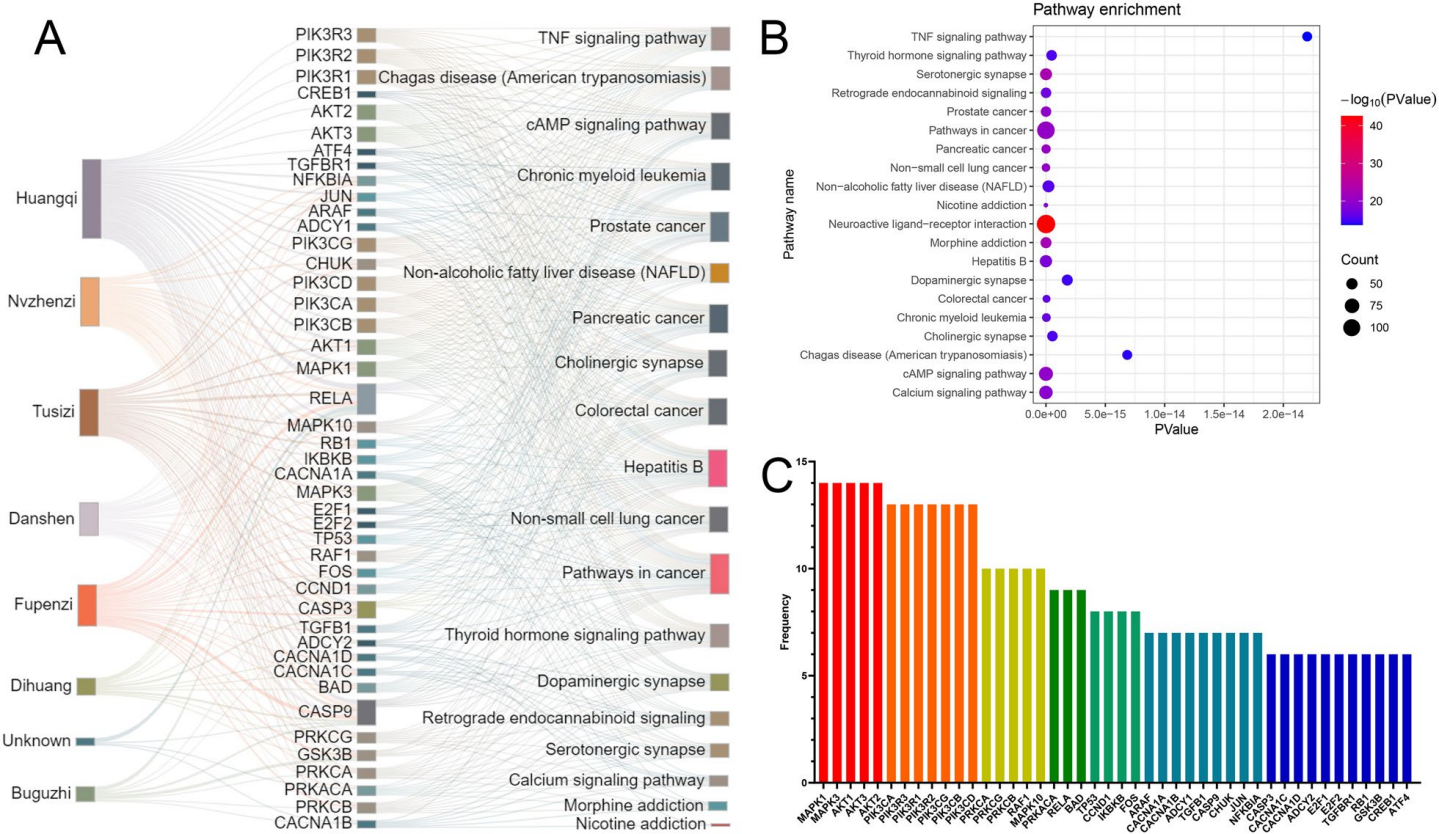
Network pharmacology analysis. **A** The network of herb-target pathways. **B** The most significant top 20 KEGG pathways. **C** The targets involved in the 20 pathways with a frequency ≥ 6

To study the complex interactions among target molecules, biological functions and the bioactive compounds of BSTJF, network pharmacology analysis was conducted. The top 20 most significant KEGG pathways are shown in Fig. 3. The neuroactive ligand-receptor interaction had the largest number of involved targets and the minimum P value. The targets involved in these 20 pathways were sorted and sequenced according to their frequency. As shown in Fig. 3, MAPK1, MAPK3, AKT1, AKT3, and AKT2 were ranked first in the targets with frequencies ≥ 6. Thereafter, the herb-target-pathway network was constructed based on the top 20 most significant KEGG pathways and the involved targets and herbs to further elucidate the molecular mechanism of BSTJF. Among the involved herbs, *Astragalus membranaceus* (Fisch.) *Bge. var. mongholicus* (Bge.) Hsiao (Huangqi) had the most corresponding targets.

### The effects of BSTJF on the PCOS model rats

#### Effect of BSTJF on PCOS rat estrous cycle alternations

**Fig. 4:**
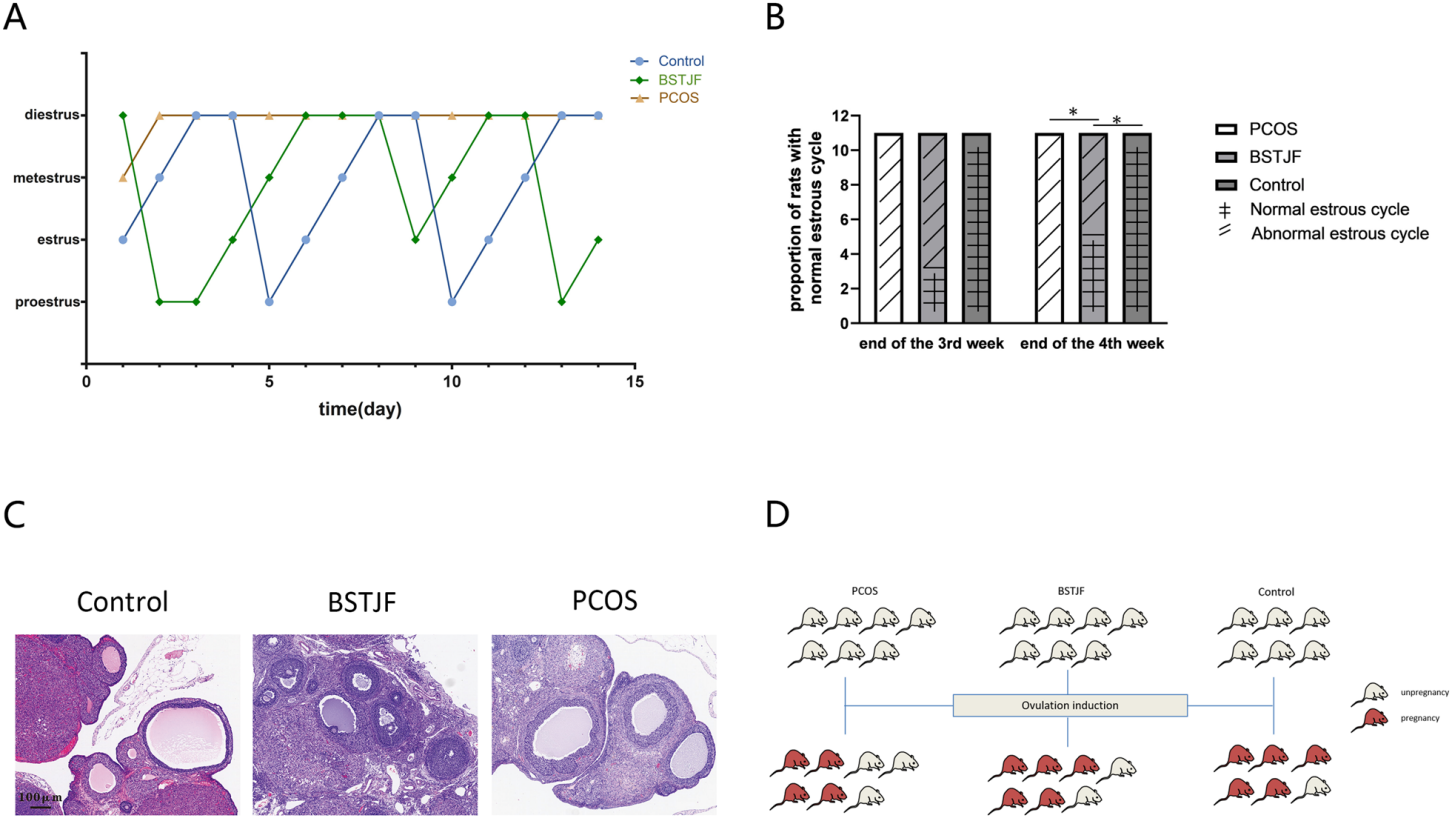
The effects of BSTJF on PCOS model rat. **A** Representative broken lines of the estrous cycle for 14 days after 2 weeks of gavage (PCOS, BSTJF and control groups). **B** The proportion of rats with normal estrous cycles at the end of the 3rd and 4th weeks of intragastric administration. The proportion of rats with normal estrous cycles is shown by bar plots. Fisher’s exact test for multiple rates comparisons (*P < 0.05). **C** Hematoxylin and eosin (H&E) staining of ovarian tissue of the PCOS, BSTJF and control groups. All scale bars are 100 μm. **D** The pregnancy of PCOS, BSTJF and control groups

The vaginal cytology of PCOS model rats exhibited an irregular estrous cycle; thus, PCOS modeling was successfully established. After the BSTJF treatment, the irregular estrous cycles of rats were reversed. Compared with that of the PCOS group, there was no difference in the proportion of rats with a normal estrous cycle in the BSTJF group at the end of the third week (P = 0.214), but there was a difference in the proportion of rats with a normal estrous cycle at the end of the fourth week (P = 0.012). (Fig. 4A, B).

#### Effect of BSTJF on the ovarian morphologic changes in PCOS rats

The control rats exhibited ovarian follicles at various stages upon hematoxylin and eosin (H&E) staining, whereas the PCOS model rats exhibited disordered ovarian morphology with evidence of cystic dilatation in the ovarian follicles. The oocytes within these follicles were absent, and there was a significant decrease in the number of granulosa cell layers. In the BSTJF group, normal tissue morphology was partially restored, with oocytes evident in follicles and an increase in the number of granulosa cell layers. (Fig. 4C).

#### Effect of BSTJF on the fertility of PCOS rats

Seven female rats each from the PCOS and BSTJF groups and six female rats from the control group were subjected to superovulation induction and cage mating. The results showed that four rats in the PCOS group and five rats in the BSTJF and control groups were pregnant. Fisher’s exact test showed that no statistically significant differences were found among groups (P > 0.05) (Fig. 4D).

#### Effect of BSTJF on MAPK3 and FOS expression in ovary granulocyte cells of PCOS rats

**Fig. 5:**
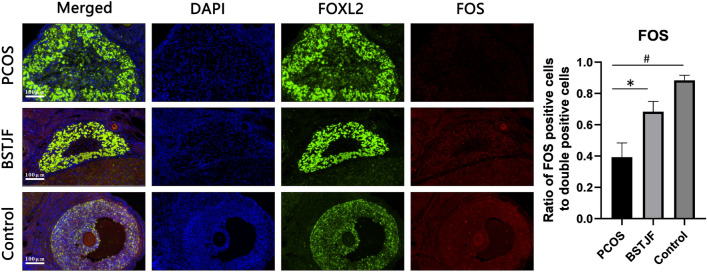
Visualization of FOS expressed in the ovarian granulosa cells of the PCOS, BSTJF and control groups by double-labeling immunofluorescence with FoxL2 (winged helix forkhead transcription factor gene2, specific expression in adult ovarian granulosa cells) (green) and FOS (red). Nuclei were counterstained with 40,6-diamidino-2-phenylindole (DAPI) (blue fluorescence). All scale bars are 100 μm. The expression ratio of the target protein is shown by bar plots (n = 4 in each group). Data were presented as the mean ± SEM. One-way ANOVA for multiple comparisons and LSD test for pairwise comparisons between groups (*P < 0.05, ^#^P < 0.01)

**Fig. 6:**
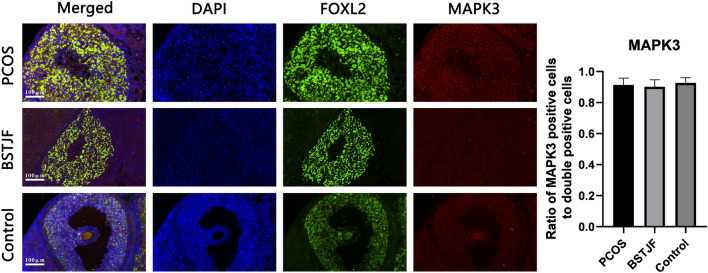
Visualization of MAPK3 expressed in ovarian granulosa cells of the PCOS, BSTJF and control groups by double-labeling immunofluorescence with FoxL2 (winged helix for head transcription factor gene2, specific expression in adult ovarian granulosa cells) (green) and MAPK3 (red). Nuclei were counterstained with 40,6-diamidino-2-phenylindole (DAPI) (blue fluorescence). All scale bars are 100 μm. The expression ratio of the target protein is shown by bar plots (n = 4 in each group). Data were presented as the mean ± SEM. One-way ANOVA for multiple comparisons and LSD test for pairwise comparisons between groups

To validate the target of network pharmacology and further explore the results of our team's preliminary study [27], target validation was focused on MAPK3 and FOS protein levels. Double-labeling immunofluorescence showed that the expression level of FOS protein in ovarian granulosa cells of the PCOS model group was reduced compared to that in the control group (P = 0.002), and BSTJF treatment significantly increased the expression level (P = 0.018). No significant difference in MAPK3 protein expression levels was found among these groups. (Figs. 5, 6).

### Alterations in the neurobehavioral manifestation and morphology of the dentate gyrus of female offspring born to PCOS rats administered BSTJF

#### Shifts in neurobehavioral performance

**Fig. 7:**
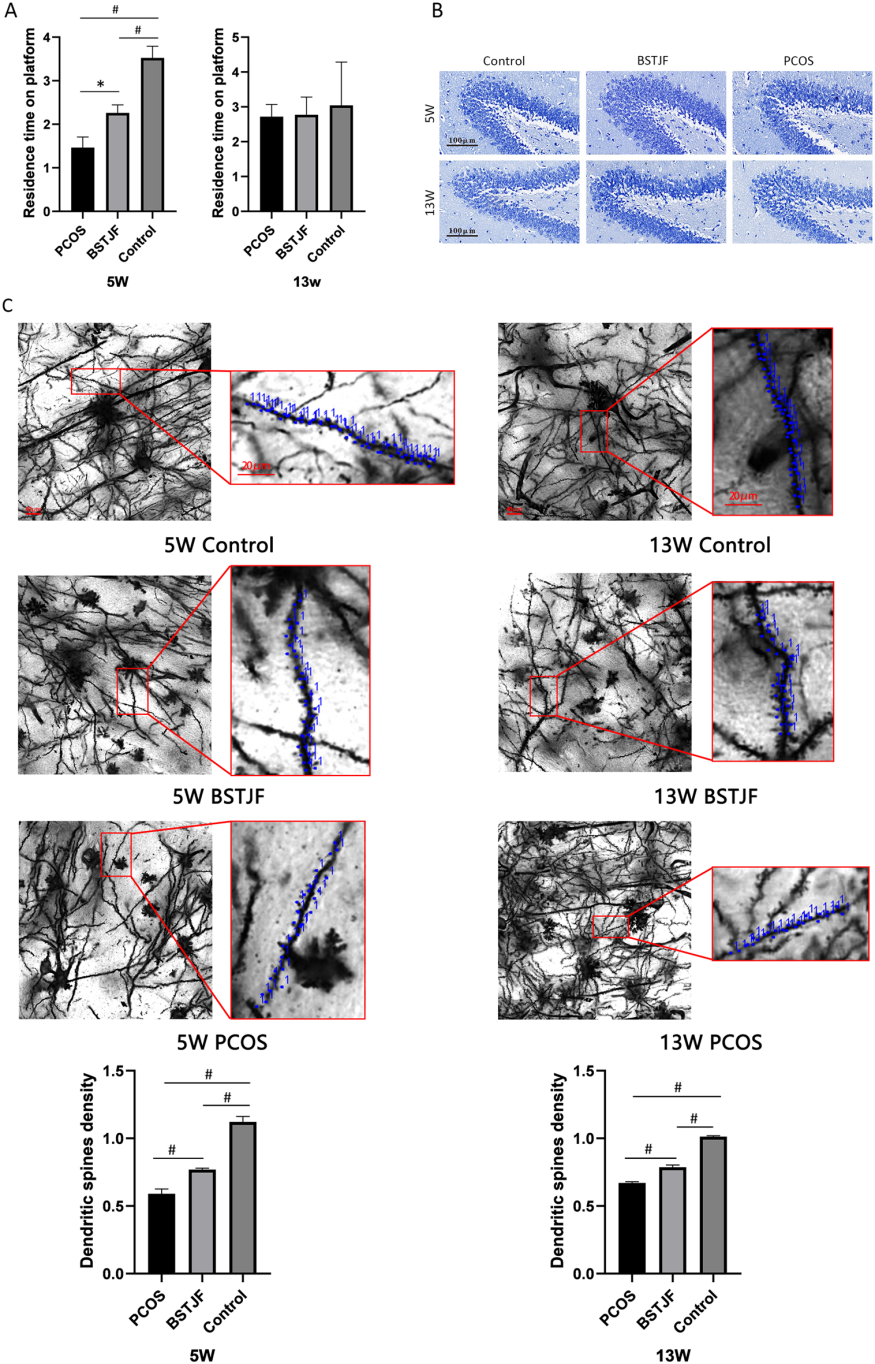
Neurobehavioral and cerebral morphological changes in the female offspring. **A** Morris water maze test in the PCOS female offspring (n = 4 in 5 weeks, n = 4 in 13 weeks), BSTJF (n = 4 in 5 weeks, n = 4 in 13 weeks) and controls (n = 4 in 5 weeks, n = 4 in 13 weeks). Data are presented as the mean ± SEM. One-way ANOVA for multiple comparisons and LSD test for pairwise comparisons between groups (*P < 0.05, ^#^P < 0.01). **B** The morphological manifestation of neurons in the dentate gyrus of female offspring by Nissl staining. The scale bars are 100 μm. **C** The morphological structure of the dendrites in the dentate gyrus of female offspring by Golgi staining. The scale bars are 40 μm. Representative dendrites are partially enlarged, and the dendritic spines are indicated in blue. The scale bars are 20 μm. The density of dendritic spines is shown by bar plots (n = 4 in each group at 5 w and 13 w). Data are presented as the mean ± SEM, one-way ANOVA for multiple comparisons and LSD test for pairwise comparisons between groups (*P < 0.05, ^#^P < 0.01)

The MORRIS water maze experiment showed that the residence time in the target zone of pubertal female offspring born to the PCOS model group was significantly lower than that of the control group (P < 0.005). In contrast, in the BSTJF group, the trend was significantly reversed when the mothers of the rats received BSTJF treatment (P = 0.039). However, no significant difference was found in the adult female offspring among the groups. (Fig. 7A).

#### Morphological observation of the hippocampus dentate gyrus

Nissl staining showed that the neurons of the dentate gyrus of female offspring had normal morphologies and were orderly in arrangement, clear in outline, and uniform in staining. In addition, the neurons had large and round nuclei, complete nuclear membranes, and obvious Nissl bodies. No significant differences were observed among the groups in either the puberty or adulthood offspring (Fig. 7B).

#### Changes in the density of dendritic spines in the dentate gyrus

Golgi staining showed that the density of dendritic spines in the dentate gyrus of the pubertal female offspring born to the PCOS model group was significantly lower than that of the control group (P < 0.005), which was restored when their mothers received BSTJF intervention (P = 0.003). These trends were still observed in the adult female offspring (P < 0.005) (Fig. 7C).

#### Ultrastructural alterations of the neurons, mitochondria and synapses in the dentate gyrus

**Fig. 8:**
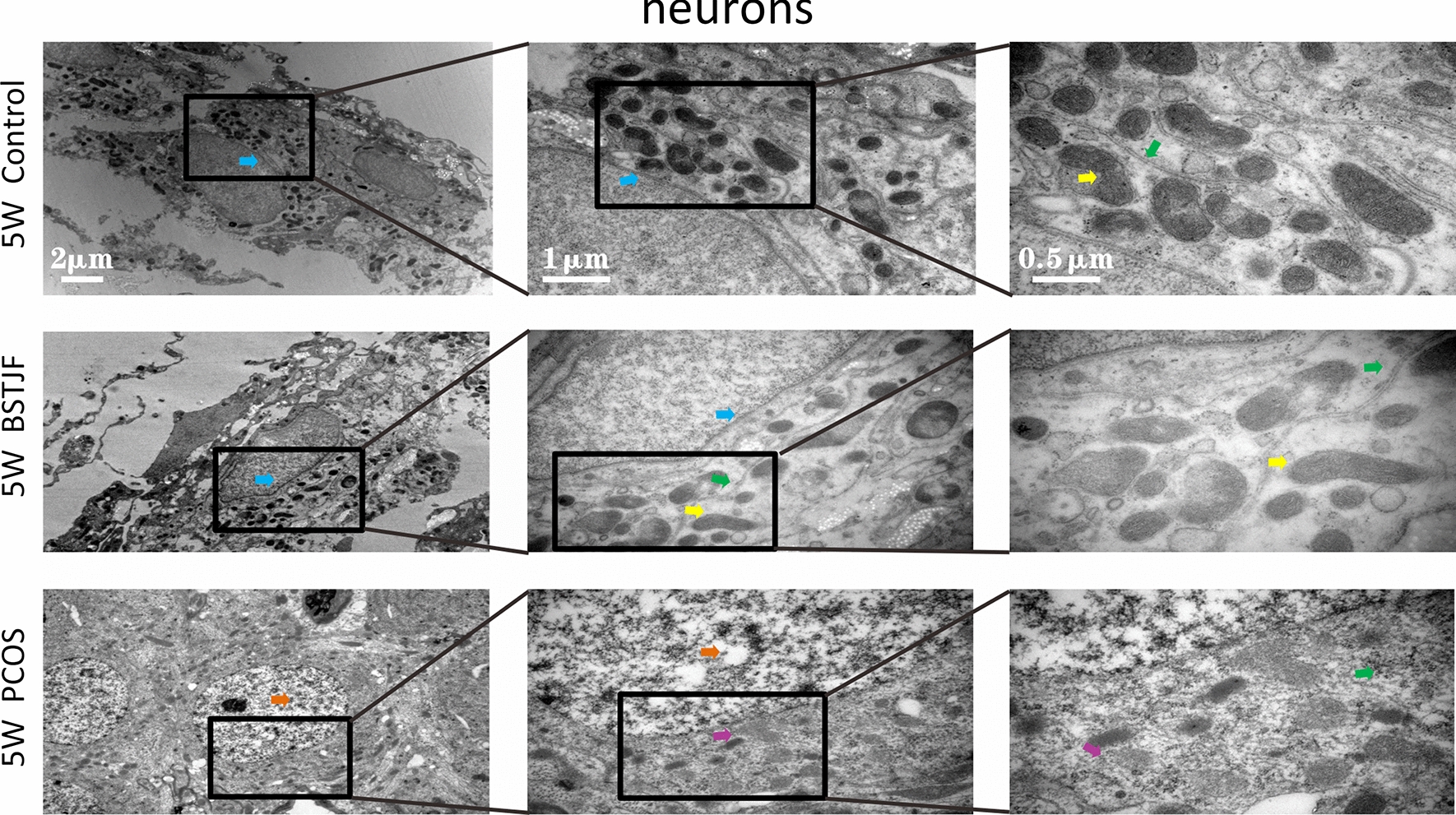
The ultrastructure of neurons in dentate gyrus of 5 w female offspring by electron microscopy (blue arrow). The nuclear chromatin was uniform, and the nuclear membrane was intact and continuous (yellow arrow). The mitochondrial form and size were normal, the bilayer membrane was intact, and the number, size and arrangement of the cristae were normal (green arrow). The rough endoplasmic reticulum was normal, and ribosomes were abundant (orange arrow). The nuclear chromatin was uneven (purple arrow). Mitochondria were swollen and dilated, while mitochondrial cristae were missing

**Fig. 9:**
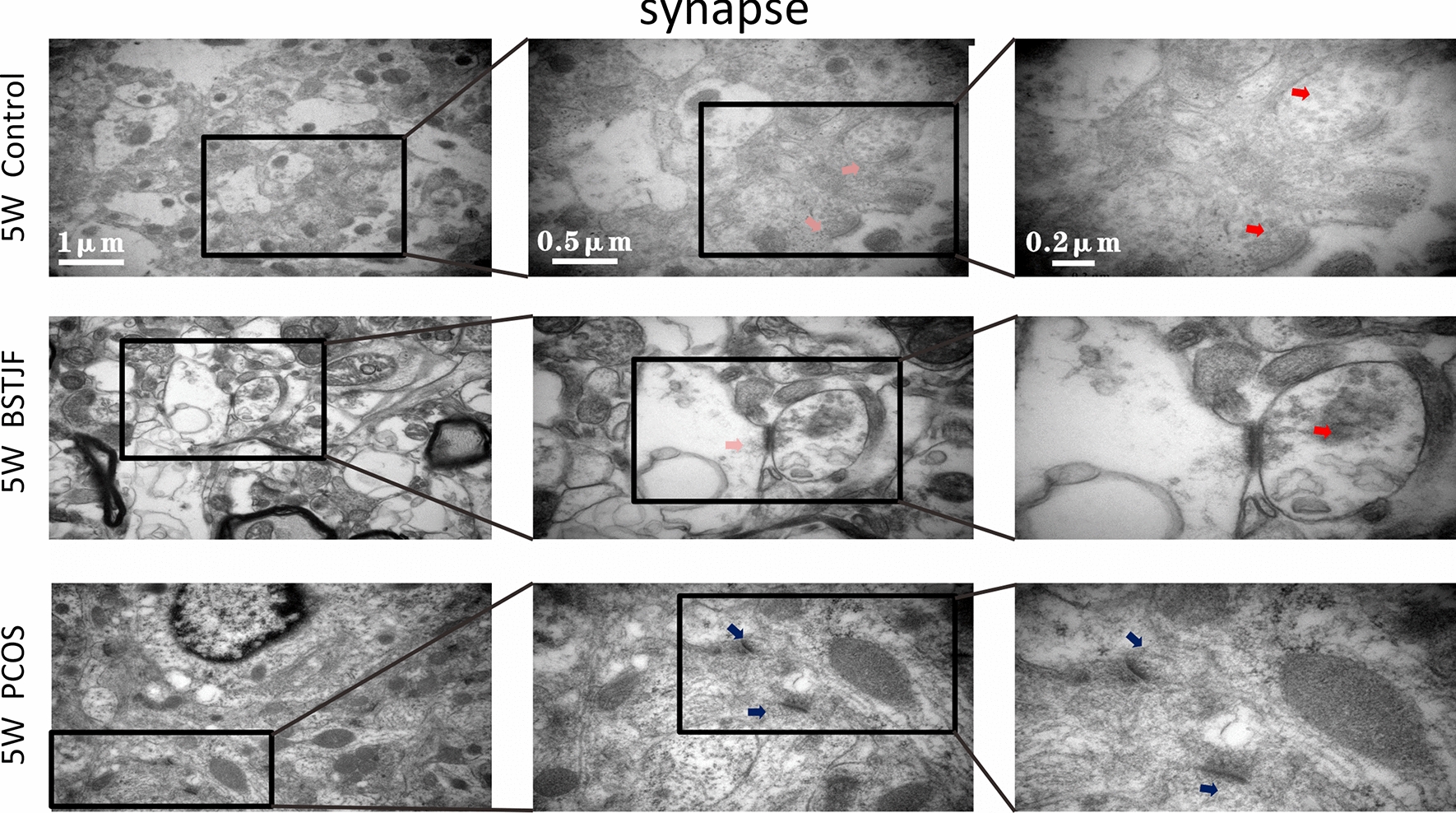
The ultrastructure of synapses in dentate gyrus of 5 w female offspring by electron microscopy (pink arrow). The synapse was long and complete and tightly knit in structure (red arrow). The synaptic vesicles were abundant (indigo arrow). The synaptic vesicles were sparse, and the synaptic clefts were blurred

**Fig. 10:**
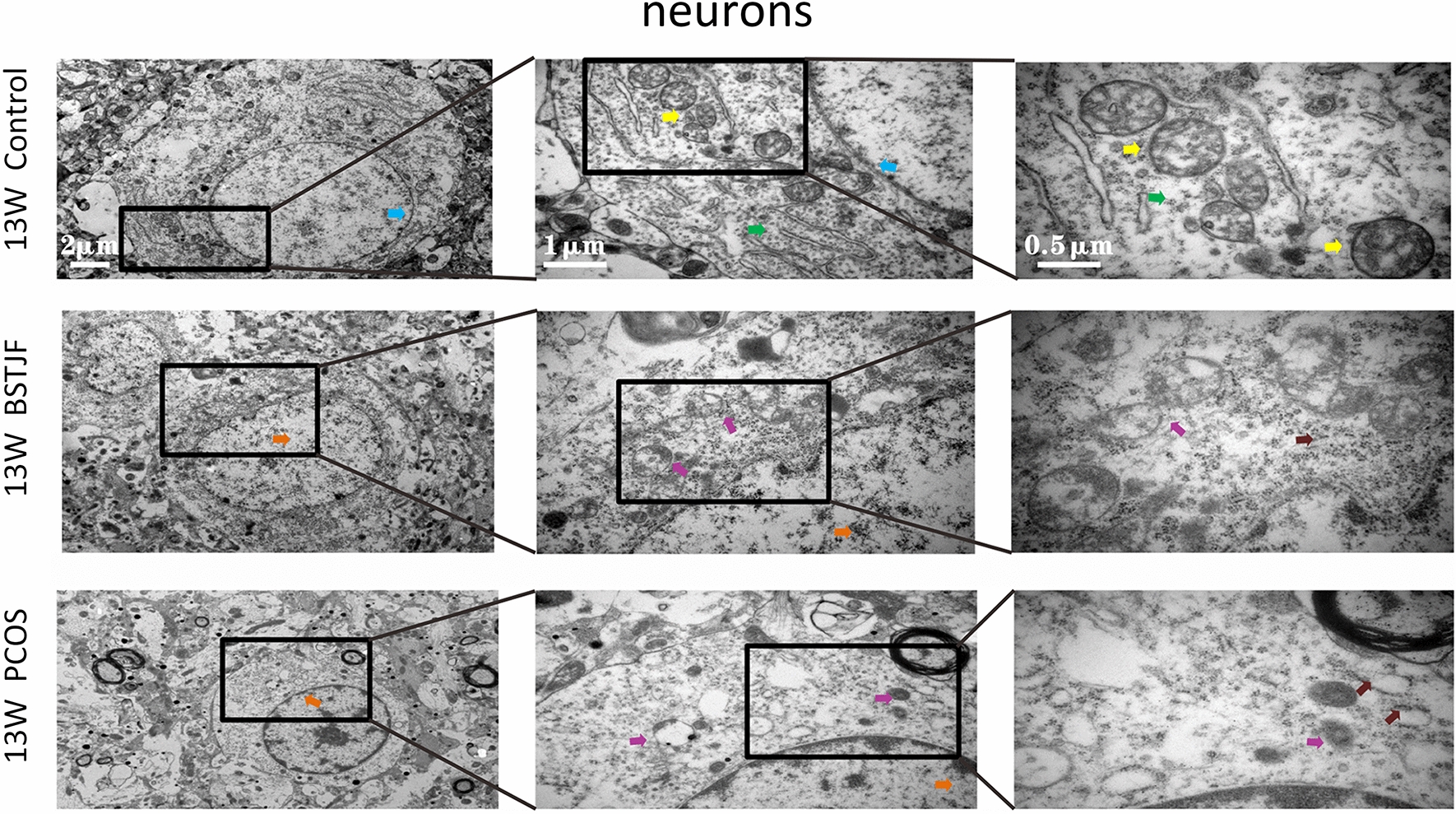
The ultrastructure of neurons in the dentate gyrus of 13 w female offspring by electron microscopy (blue arrow). The nuclear chromatin was uniform, and the nuclear membrane was intact and continuous (yellow arrow). The mitochondrial form and size were normal, the bilayer membrane was intact, and the number, size and arrangement of the cristae were normal (green arrow). The rough endoplasmic reticulum was normal, and ribosomes were abundant (orange arrow). The nuclear chromatin was uneven (purple arrow). Mitochondria were swollen and dilated, while mitochondrial cristae were missing (brown arrow). The rough endoplasmic reticulum was decreased, and local ribosomes fell off

**Fig. 11:**
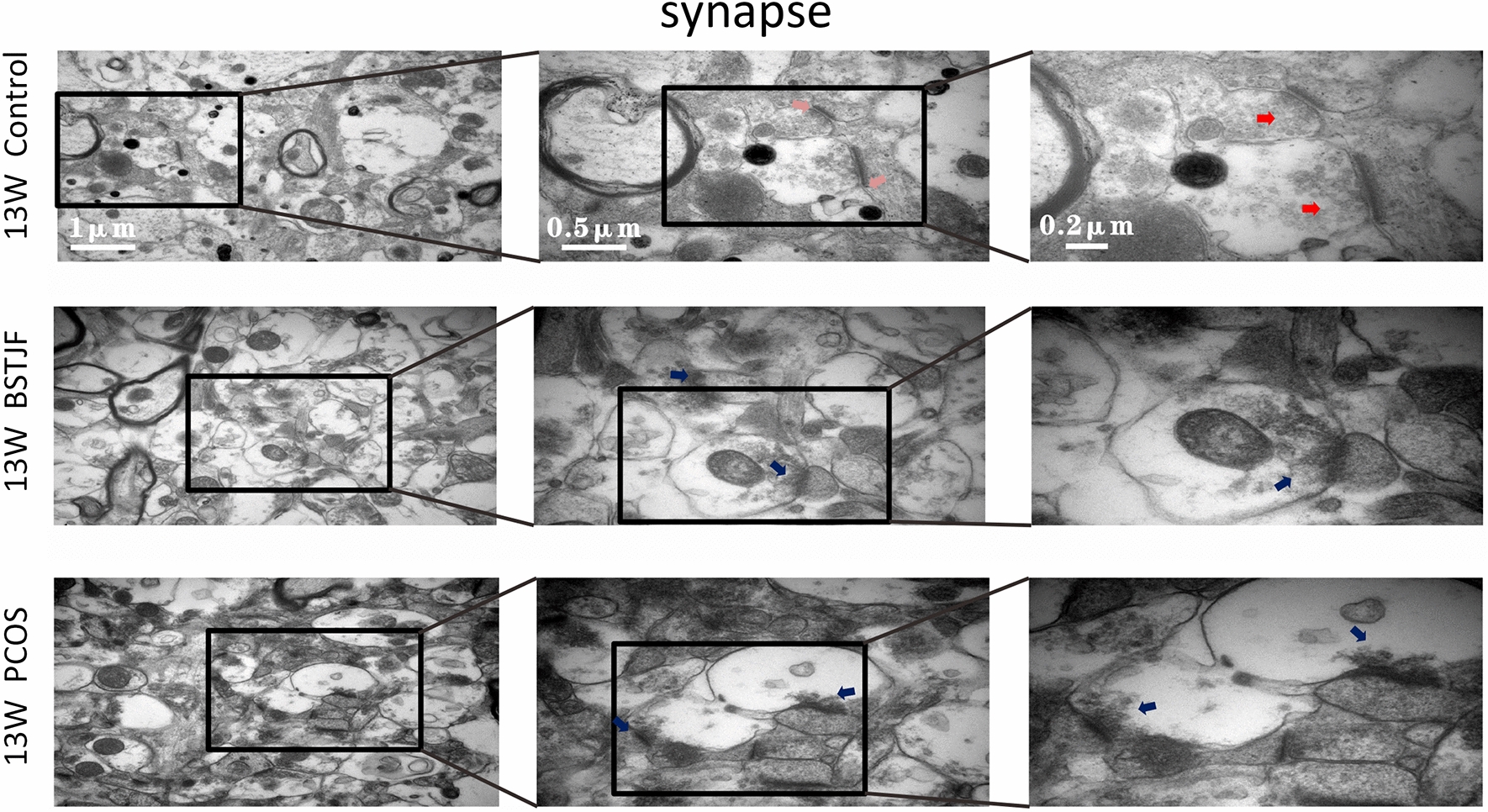
The ultrastructure of synapses in dentate gyrus of 13 w female offspring by electron microscopy (pink arrow). The synapse was long and complete and tightly knit in structure (red arrow). The synaptic vesicles were abundant (indigo arrow). The synaptic vesicles were sparse, and the synaptic clefts were blurred

Transmission electron microscopy showed that the nuclei of the neurons in the dentate gyrus in the pubertal female offspring born to PCOS model rats were uneven. The cytoplasm was partially dissolved. Moreover, the mitochondria were swollen and dilated with ruptured mitochondrial cristae, the rough endoplasmic reticulum was reduced, and the synaptic structure was partially dissolved with aberrant synaptic vesicles and blurred synaptic clefts. In progeny born to the BSTJF group, these negative changes were altered, whereas the reversion in adulthood was not obvious (Figs. 8, 9, 10, 11).

#### Transformation of Gabrb1, Grin2b and Adra1b expression levels in the dentate gyrus detected by immunohistochemistry

**Fig. 12:**
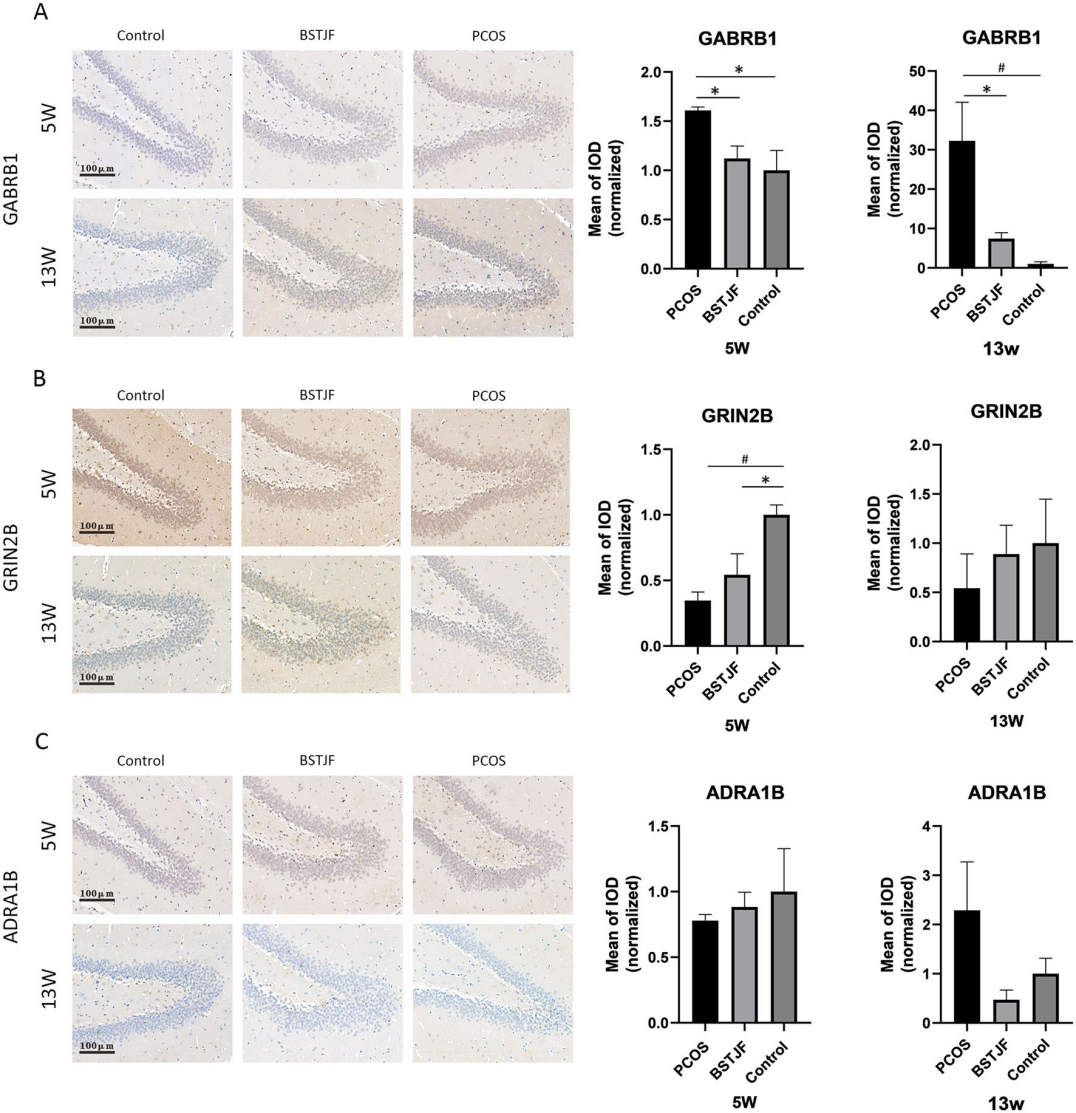
Gabrb1 (**A**), Grin2b (**B**) and Adra1b (**C**) protein expression levels in the dentate gyrus of the female offspring of PCOS, BSTJF and controls, as assessed by immunohistochemical staining. The scale bars are 100 μm. The bar plot shows the mean integrated optical density (IOD) (n = 4 in each group at 5 w and 13 w). Data were presented as the mean ± SEM. One-way ANOVA for multiple comparisons and LSD test for pairwise comparisons between groups. (*P < 0.05, ^#^P < 0.01)

The immunohistochemical results showed that the expression level of Gabrb1 protein in the dentate gyrus region of pubertal female offspring born to PCOS was significantly increased compared to that of the control group (P = 0.013), while the Grin2b protein expression level was significantly decreased (P = 0.002). The expression level of Gabrb1 (P = 0.016) was decreased in the case of BSTJF treatment received by their mothers, but the tendency of Grin2b protein did not change (Fig. 12A, B).

In adult female offspring, Gabrb1 protein expression was significantly higher in the model group than in the control group (P = 0.004) and was reduced in the BSTJF intervention group (P = 0.014). However, there was no significant difference in Grin2b protein expression levels among the adult female offsprings (Fig. 12A, B).

There was no significant difference in the expression level of the Adra1b protein in the dentate gyrus of the female offspring during puberty or adulthood. (Fig. 12C).

#### Transformation of Gabrb1, Grin2b and Adra1b expression levels in the dentate gyrus detected by western blotting

**Fig. 13:**
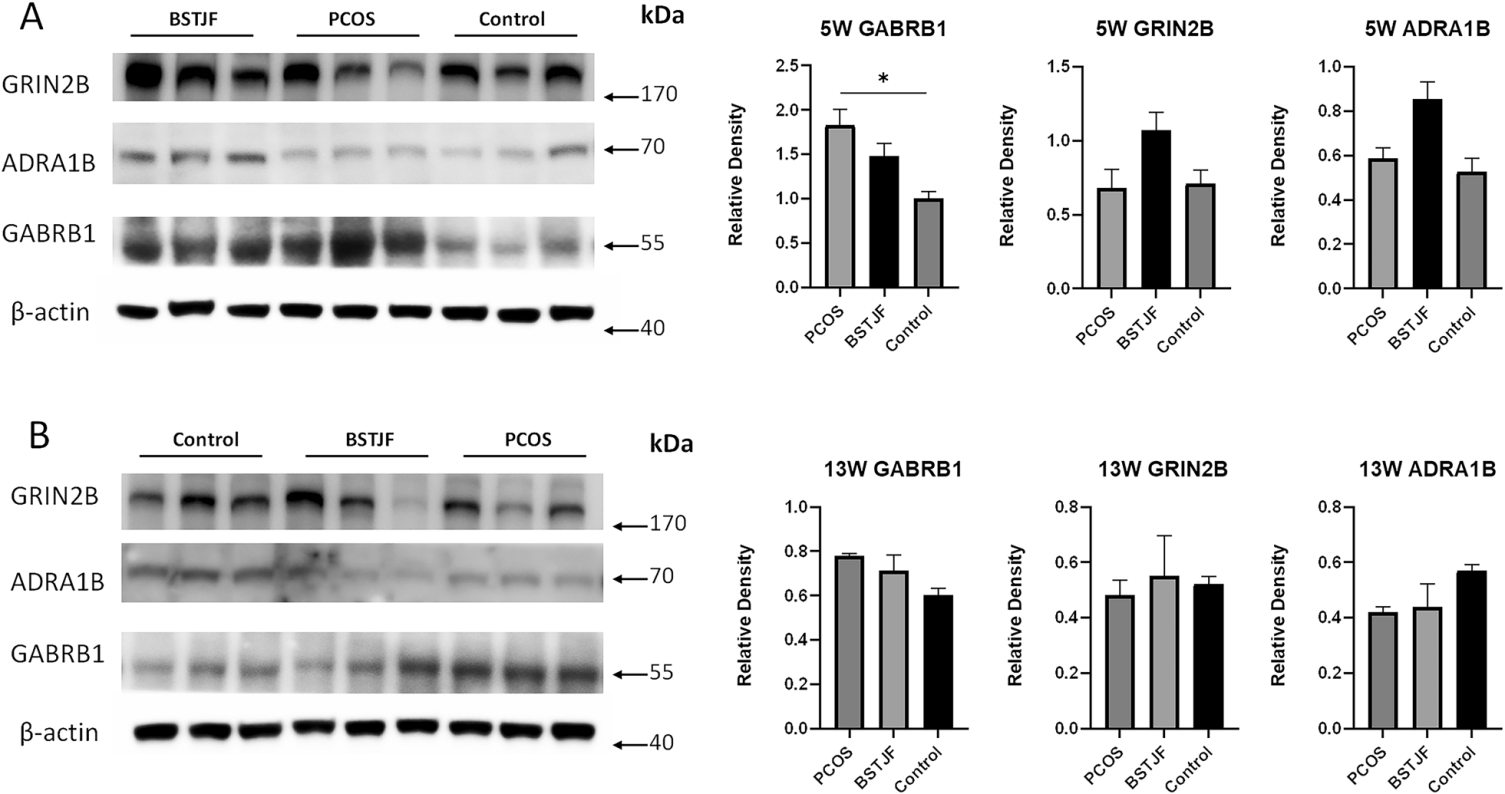
Gabrb1, Grin2b and Adra1b protein expression levels in the hippocampus of 5 w (**A**) and 13 w (**B**) female offspring of PCOS, BSTJF and control groups, as assessed by Western Blotting. The bar plot shows the relative density of gray values of the target protein strips (n = 3 in each group at 5 w and 13 w). Data were presented as the mean ± SEM. Non-parametric test for multiple comparisons and pairwise comparisons between groups. (*P < 0.05)

The protein expression level of Gabrb1 in the hippocampus of pubertal female offspring born to PCOS rats was significantly increased compared to that of the control group (P = 0.034), while no significant differences were found in the expression levels of Grin2b and Adra1b. Although the expression level of Gabrb1 had the tendency to decrease in BSTJF group, the difference was not statistically significant (Fig. 13A). For the adult female offspring, no significant differences were found in the protein expression levels of Gabrb1, Grin2b and Adra1b (Fig. 13B).

## Discussion

In the past few decades, oral contraceptive pills (OCPs), clomiphene citrate, and letrozole have been widely used in PCOS patients [43–45]. However, there have been reports regarding the side effects and limitations of existing pharmaceutical agents [5, 46]. The literature shows that Chinese medicine has a positive effect on PCOS, which can target reproductive and metabolic defects, regulate abnormal hormone levels, and reduce related mental stress [5–7]. Chinese medicine has been found to improve the autism-like behavior of offspring caused by prenatal DHT exposure by inhibiting androgen receptors [31] and reduce growth retardation and behavioral changes caused by "fetal damage" in pregnant rats [32].

However, most TCM formulas were obtained from empiricism, leading to the problem that although their efficacy has been validated in clinical practice, they lack the support of scientific and rational mechanisms [47]. TCM generally exerts its therapeutic effect through the characteristics of multiple components, target, and channels, which are achieved on the basis of its chemical constituents [48]. Mass spectrometry (MS) and molecular networking (MN) based on MS and network pharmacology have been widely used in the study of chemical constituents of traditional Chinese medicine [23, 49, 50]. MN can be used to visually show all the molecular ions detected in a complete LC–MS/MS experiment and the chemical relationships among them [51] and to perform qualitative and quantitative analysis of compounds from various sources [52]. Network pharmacology can generate complicated interaction networks based on target molecules, biological functions, and bioactive compounds, which meet the natural characteristics of TCM formulas and enable the systematic elucidation of the mechanism of action of TCM formulas at the molecular level [53]. When the strategy is applied in pharmacological research of TCM formulas, new opportunities are provided for the discovery of bioactive compounds, mechanistic research, quality control, and many other fields [50]. The application of network pharmacology in TCM formulas is expected to change from experience-based medicine to evidence-based medicine.

In the network pharmacology section of this study (Figs. 1, 2, 3), a total of 91 constituents were characterized using the BSTJ formula. Among these, 30 compounds were identified by comparison with reference standards in retention time and mass spectra. The 20 most significant KEGG pathways and the high-frequency genes of these pathways were predicted to be putative targets of these molecules. The results showed that the neuroactive ligand-receptor interaction had the largest number of involved targets, and MAPK1 and MAPK3 were ranked first among the targets with a frequency ≥ 6.

A pathway study illustrated that the neuroactive ligand-receptor interaction pathway plays a role in the regulation of egg production in chickens [54]. Moreover, He et al. found that the differentially expressed genes of PCOS were enriched in the neuroactive ligand-receptor interaction pathway [55]. The MAPK3/1 (epidermal growth factor receptor/extracellular signal-regulated kinase (ERK) 1/2) signaling cascade plays an essential role in ovulation [56, 57]. Therefore, we used animal models to test whether there were intergroup differences in the expression level of MAPK3 in ovarian granulosa cells to explore whether BSTJF affected PCOS rats through related pathways (Figs. 4, 6). Since no difference in MAPK3 expression was found in this study, we hypothesized that BSTJF may play a role in other ways.

On the other hand, FOS, which had been shown to be differentially expressed in granulosa cells of PCOS rats by our previous research [27], is also one of the targets with a frequency ≥ 6 in the results of the network pharmacology section (Fig. 3). In humans, FOS expression levels are downregulated in the adipose tissue of PCOS patients [58] and placental villi [27]. Furthermore, it was upregulated in granulosa cells collected from in vitro fertilization (IVF) patients treated with human chorionic gonadotropin (HCG) compared with those isolated before administration [59]. Furthermore, FOS-deficient mice failed to ovulate even when exogenous gonadotropins were administered, indicating that ovarian expression of FOS is necessary for ovulation [60]. We found the difference in the expression level of FOS between the PCOS group and the control group was reversed in the BSTJF group, which gave us reason to suppose that BSTJF may act through a FOS-related pathway.

Since PCOS can affect the nervous system of the offspring, we further investigated whether the neurobehavioral performance of the offspring of BSTJF interfered rats would be upturn, and the results were positive (Figs. 7, 8, 9, 10, 11). On the other hand, our previous transcriptomic study [27] showed that Gabrb1, Grin2b and Adra1b, which were enriched in the neuroactive ligand-receptor interaction pathway, were differentially expressed in the hippocampus of PCOS progenies. Therefore, we examined the expression levels of these three target proteins in the hippocampus of progeny rats in the PCOS, BSTJF and control groups to explore whether BSTJF acts on progeny through related pathways (Figs. 12, 13).

The FOS-dependent neuronal ensemble was found to promote memory generalization, and isolated rats showed anxiety-like behavior, worse working memory and lower levels of learning-related FOS immunoreactivity [61, 62]. Gabrb1, the gene encoding the β1 subunits of GABA receptor type A (GABAAR), has been confirmed to be associated with intelligence [63], working memory performance [64], antipsychotic dosage [65], and addictive behavior [66]. GABAAR is the target of agonist inhibitors and convulsive antagonists and the other (allosteric) binding sites of most GABAAR drugs on GABAAR proteins [67]. The Grin2b gene plays a crucial role in normal neuronal development and is important for learning and memory [68]. The expression of Grin2b/NR2B was significantly decreased in the hippocampus of adolescent offspring in the prenatal hypoxia-induced learning and memory disability group [69]. It is also related to neurodevelopmental disorders, such as developmental delay/intellectual disability, muscle tone abnormalities, epilepsy, and autism spectrum disorder [70].

Based on the above results, although the change in FOS expression level in parental ovarian granulosa cells could not be directly proven to be related to the changes in Gabrb1 and Grin2b expression levels in the hippocampus of offspring, it is reasonable to speculate that BSTJF may improve neurobehavioral performance across generations through pathways related to these proteins. As ovarian granulosa cells play indispensable roles in ensuring the production of fully differentiated oocytes that can give rise to healthy embryos [71], changes in the expression level of FOS in ovarian granulosa cells may affect the development of the offspring nervous system by changing the developmental environment of oocytes or by other unknown pathways, which is worthy of further study.

In addition, this study explored the effect of BSTJF on the neurological alteration of female offspring born to PCOS model rats. As the dentate gyrus in the hippocampus has an essential role in spatial behavior [72] and the depression, anxiety and stereotypical behavior of offspring caused by androgen exposure during pregnancy may also be related to changes in the dentate gyrus [73], our morphological observation focused only on the dentate gyrus. BSTJF affects the behavior of offspring at 5 weeks of age and these effects disappeared at 13 weeks of age; however, the effects on the expression of hippocampal dendritic spines and related receptor proteins were still present. The literature has shown that GABA receptors are related to learning, memory, and neurobehaviors such as anxiety and depression [74, 75]. Therefore, the influence of BSTJF on adult offspring may be reflected in other aspects, such as anxiety/depression, which is worth further exploration.

This study has several limitations regarding network pharmacology and animal experiments. Despite its broad prospects, network pharmacology still has several limitations. First, the existing database was incomplete. Second, with the popularization of network pharmacology technology, different model algorithms have been developed, and different algorithms produce different prediction results; therefore, it is necessary to select an appropriate algorithm for different purposes to ensure the accuracy of the results. Third, the application of network pharmacology in Chinese medicine formula research is mainly in the qualitative stage, whether determining new targets or drug mechanism research. However, there is a dose-efficacy relationship between the drugs and disease, and current network pharmacology technology is difficult to quantify. Last, most studies based on network pharmacology are still on static network analysis, whereas body function is an ongoing dynamic process, and the occurrence of disease, development, and efficacy of drugs are also dynamic changes. Therefore, many experimental verifications in vivo or in vitro are needed [47]. For the rat experiment, the sample size of rats randomly selected for neurobehavioral manifestation testing was relatively small to minimize the influence of the estrous cycle on behavioral tests of female rats, and more samples are needed to confirm the present conclusions. In addition, the PCOS rat model used here represented a restricted range of PCOS phenotypes in women. Furthermore, there are significant differences between human and animal models in assessing learning and memory because human upbringing is difficult to reflect in animal models.

## Conclusion

BSTJF can effectively improve ovarian follicle development in PCOS rats by increasing FOS protein expression levels in ovarian granulosa cells and has positive effects on pubertal neurobehavioral alterations in female offspring born to these rats by reversing dendritic spine density, the ultrastructure of neurons and synapses, and Gabrb1 and Grin2b protein expression levels in the hippocampus.

## Supplementary Information

**Additional file 1: Table S1.** Compounds identified from BSTJF by LC-Q-TOF-MS. **Figure S1.** Mass spectrum chromatograms of representative reference standards.

## Data Availability

The datasets used and/or analyzed during the current study are available from the corresponding author on reasonable request.

## Abbreviations

CHM: Chinese herbal medicine
LC–MS: Liquid chromatography coupled with mass spectrometry
PCOS: Polycystic ovarian syndrome
BSTJF: Bu-Shen-Tian-Jing Formula
RT: Room temperature
