# Supplementary material for: Neurobehavioral alternations of the female offspring born to polycystic ovary syndrome model rats administered by Chinese herbal medicine
Source: Chin Med. 2021 Oct 2;16:97. doi: 10.1186/s13020-021-00512-4 (PMC8487466; doi:10.1186/s13020-021-00512-4)
Supplement: Supplementary file 1 — Additional file 1: Table S1. Compounds identified from BSTJF by LC-Q-TOF-MS. Figure S1. Mass spectrum chromatograms of representative reference standards. [file 13020_2021_512_MOESM1_ESM.docx]

Supplementary Table 1. Compounds identified from BSTJF by LC-Q-TOF-MS

| No. | RT | Negative ion | Positive ion | Molecular Formula | Identity | Source |
| --- | --- | --- | --- | --- | --- | --- |
| 1 | 1.12 | 195.0519 |  | C_6_H_12_O_7_ | Gluconic acid |  |
| 2 | 1.20 | 503.1610 |  | C_18_H_32_O_16_ | Planteose | DS |
| 3 | 1.53 | 341.1087 |  | C_12_H_22_O_11_ | Sucrose* | DH、HQ |
| 4 | 1.83 | 665.2152 |  | C_24_H_42_O_21_ | Stachyose | DH |
| 5 | 4.46 | 355.1240 |  | C_13_H_24_O_11_ | Methyl β-D-lactopyranoside |  |
| 6 | 5.49 | 407.1191 |  | C_15_H_22_O_10_ | Catalpol* | DH |
| 7 | 6.50 | 603.1773 |  | C_22_H_36_O_19_ | Aldotetraouronic acid |  |
| 8 | 6.61 |  | 268.1040 | C_10_H_13_N_5_O_4_ | Adenosine* | HQ |
| 9 | 6.82 | 429.1245 |  | C_14_H_24_O_12_ | 6-O-acetyl sucrose |  |
| 10 | 7.82 |  | 166.0860 | C_9_H_11_NO_2_ | L-tyrosine |  |
| 11 | 8.60 | 495.1356 |  | C_18_H_26_O_13_ | 3-hydroxy-5-methoxyphenol-2-O-beta-apiofuranosyl-(1->2)-beta-glucopyranoside | HQ |
| 12 | 10.47 | 197.0466 |  | C_9_H_10_O_5_ | Danshensu* | DS |
| 13 | 10.50 | 395.0981 |  | C_18_H_20_O_10_ | Reaxys ID: 25324304 |  |
| 14 | 13.20 | 731.2258 |  | C_27_H_42_O_20_ | Rehmannioside D* | DH |
| 15 | 14.52 | 315.1084 |  | C_14_H_20_O_8_ | β-(3,4-dihydroxyphenyl)-ethyl-O-β-D-glucopyranoside | NZZ |
| 16 | 14.70 | 137.0262 |  | C_7_H_6_O_3_ | 3,4-Dihydroxybenzaldehyde* | DS |
| 17 | 14.78 | 373.1136 |  | C_16_H_22_O_10_ | Geniposidic acid | DH |
| 18 | 15.87 | 375.129 |  | C_16_H_24_O_10_ | 8-epiloganic acid | DH |
| 19 | 15.95 | 461.1289 |  | C_19_H_26_O_13_ | Ligulucidumoside B | NZZ |
| 20 | 16.05 | 353.0869 |  | C_16_H_18_O_9_ | Neochlorogenic Acid* | TSZ |
| 21 | 17.03 | 213.0766 |  | C_10_H_14_O_5_ | Pentanedioic acid | NZZ |
| 22 | 18.49 | 341.0872 |  | C_15_H_18_O_9_ | 6-O-caffeoyl-D-glucopyranoside | DS |
| 23 | 18.57 | 299.1134 | 301.1287 | C_14_H_20_O_7_ | Salidroside* | NZZ、DH、  DS |
| 24 | 19.43 | 433.0985 |  | C_17_H_24_O_14_ | Nuezhenidic Acid* | NZZ |
| 25 | 20.11 | 419.1190 |  | C_17_H_24_O_12_ | 3-O-[beta-D-apiofuranosyl-(1→2)-beta-D-glucopyranosyl]maltol | HQ |
| 26 | 24.63 | 353.0877 | 355.1034 | C_16_H_18_O_9_ | Chlorogenic acid* | TSZ、NZZ、HQ、DS |
| 27 | 27.53 | 389.1082 |  | C_16_H_22_O_11_ | (-)-secologanoside | NZZ |
| 28 | 28.83 | 291.0142 | 293.0298 | C_13_H_8_O_8_ | Brevifolin carboxylic acid | FPZ |
| 29 | 31.32 | 403.1236 |  | C_17_H_24_O_11_ | 8-Epiisokingiside | NZZ |
| 30 | 34.16 | 163.0410 |  | C_9_H_8_O_3_ | p-Coumaric Acid* | TSZ、NZZ、FPZ、DH、  HQ、DS |
| 31 | 34.77 | 487.1443 |  | C_21_H_28_O_13_ | Cistanoside F | DH |
| 32 | 35.57 | 183.1035 |  | C_10_H_16_O_3_ | 1. hydroxy-2,6,6-trimethyl-1-   cyclohexene-1-carboxylic acid | DH |
| 33 | 38.39 | 365.0875 |  | C_17_H_18_O_9_ | Psoralenoside* | BGZ |
| 34 | 41.53 | 223.0618 |  | C_11_H_12_O_5_ | Sinapinic acid | FPZ |
| 35 | 41.60 | 365.0876 |  | C_17_H_18_O_9_ | Isopsoralenoside* | BGZ |
| 36 | 42.94 | 387.1293 |  | C_16_H_22_O_8_ | Coniferin | HQ |
| 37 | 45.08 |  | 417.1193 | C_21_H_20_O_9_ | Daidzin | BGZ |
| 38 | 46.32 | 381.1917 |  | C_20_H_30_O7 | 3β,5,14-trihydroxy-5β,14β  -estrane-10,17α-dicarboxylic acid |  |
| 39 | 48.50 | 785.2512 |  | C_35_H_46_O_20_ | Purpureaside B/echinacoside | DH |
| 40 | 49.08 | 563.1399 | 565.1573 | C_26_H_28_O_14_ | Schaftoside* | DS |
| 41 | 51.52 | 595.1292 | 597.1476 | C_26_H_28_O_16_ | Quercetin-O-hexoside-O-pentoside |  |
| 42 | 51.54 | 491.118 |  | C_22_H_22_O_10_ | Calycosin-7-O-Beta-D-Glucoside* | HQ |
| 43 | 52.16 | 300.9990 |  | C_14_H_6_O_8_ | Ellagic Acid | FPZ |
| 44 | 52.84 | 463.0872 | 465.1044 | C_21_H_20_O_12_ | Hyperoside* | TSZ、FPZ、NZZ、HQ |
| 45 | 53.38 | 609.1449 | 611.1632 | C_27_H_30_O_16_ | Rutin* | TSZ、NZZ、FPZ、DH、  DS |
| 46 | 55.95 | 623.1966 |  | C_29_H_36_O_15_ | Acteoside* | DH、NZZ |
| 47 | 56.92 | 593.1494 | 595.1681 | C_27_H_30_O_15_ | 7,4'-di-O-(β-D-glucopyranosyl)  apigenin/kaempferol-3-rutinoside | DS/FPZ |
| 48 | 57.40 | 523.1803 |  | C_25_H_32_O_12_ | 6-O-(E)-feruloyl ajugol | DH |
| 49 | 57.42 | 417.0817 |  | C_20_H_18_O_10_ | Salvianolic Acid D* | DS |
| 50 | 57.42 | 685.2335 |  | C_31_H_42_O_17_ | Specneuzhenide* | NZZ |
| 51 | 57.43 |  | 704.2793 | C_24_H_46_O_22_ | Lactitol sugar |  |
| 52 | 57.53 |  | 287.0557 | C_15_H_10_O_6_ | Luteolin | TSZ、NZZ、HQ、DS |
| 53 | 57.53 | 447.0921 | 449.1095 | C_21_H_20_O_11_ | Astragalin* | HQ、TSZ、  NZZ、DS |
| 54 | 57.64 | 623.1971 |  | C_29_H_36_O_15_ | Isoacteoside | DH、NZZ |
| 55 | 59.49 | 359.0768 |  | C_18_H_16_O_8_ | Rosmarinic acid* | DS |
| 56 | 59.54 |  | 533.1312 | C_25_H_24_O_13_ | 6''-O-malonate-calycosin-7-O-β-D-glucoside | HQ |
| 57 | 60.12 | 685.2340 |  | C_31_H_42_O_17_ | Nuzhenide/isonuezhenide | NZZ |
| 58 | 60.94 | 537.1026 |  | C_27_H_22_O_12_ | Lithospermic acid | DS |
| 59 | 60.97 | 493.1126 |  | C_26_H_22_O_10_ | Isosalvianolic acid A or iosmer | DS |
| 60 | 61.00 | 475.1233 | 431.1353 | C_22_H_22_O_9_ | Ononin* | HQ |
| 61 | 62.32 |  | 489.1408 | C_24_H_24_O_11_ | 6"-O-acetylcalycosin-7-O-β-D-glucopyranoside | HQ |
| 62 | 62.69 | 519.0918 | 521.1100 | C_27_H_20_O_11_ | Melitric acid A or isomer |  |
| 63 | 62.72 | 717.1292 | 719.1644 | C_36_H_30_O_16_ | Salvianolic Acid B* | DS |
| 64 | 63.13 | 493.1126 |  | C_26_H_22_O_10_ | isosalvianolic acid A or iosmer | DS |
| 65 | 63.16 | 353.1025 | 355.1187 | C_20_H_18_O_6_ | Erythrinin C | BGZ |
| 66 | 63.29 |  | 301.1081 | C_17_H_16_O_5_ | (2S)-6-methoxy-7-hydroxymethyl-4'-hydroxyflavanone | BGZ |
| 67 | 63.35 | 501.1027 |  | C_24_H_22_O_12_ | Feruloyltartaric acid/  diferuloyl-tartaric acid |  |
| 68 | 63.78 |  | 187.0387 | C_11_H_6_O_3_ | Psoralen* | BGZ |
| 69 | 64.12 | 717.1449 |  | C_36_H_30_O_16_ | Salvianolic Acid E* | DS |
| 70 | 64.13 | 519.0918 | 521.1099 | C_27_H_20_O_11_ | Melitric acid A or isomer |  |
| 71 | 64.20 | 301.0350 |  | C_15_H_10_O_7_ | Quercetol* | FPZ、NZZ、HQ、TSZ |
| 72 | 64.37 |  | 297.0765 | C_17_H_12_O_5_ | 1. ((4-oxo-2-phenyl-4H-chromen-7   -yl)oxy)acetic acid |  |
| 73 | 64.39 | 283.068 |  | C_16_H_12_O_5_ | Calycosin* | HQ |
| 74 | 64.39 | 493.1128 |  | C_26_H_22_O_10_ | Salvianolic Acid A* | DS |
| 75 | 64.61 | 1071.3553 |  | C_48_H_64_O_27_ | Liguside A/Liguside B | NZZ |
| 76 | 65.09 | 519.1492 | 521.1671 | C_25_H_28_O_12_ | 6'-O-trans-Cinnamoyl iso-8-epikingisidic acid | NZZ |
| 77 | 65.16 |  | 517.1357 | C_25_H_24_O_12_ | Kaempferol-3-O-(3,4-di-O-acetyl-α-L-rhamnopyranoside) |  |
| 78 | 65.16 |  | 187.0388 | C_11_H_6_O_3_ | Isopsoralen* | BGZ |
| 79 | 65.17 | 267.0663 |  | C_16_H_12_O_4_ | 1,2-bis(4-carboxyphenyl)ethene |  |
| 80 | 65.31 | 593.1287 | 595.1468 | C_30_H_26_O_13_ | Kaempferol 3-O-(2''-O-p-trans-coumaroyl)-beta-D-glucopyranoside/tiliroside | FPZ |
| 81 | 65.43 |  | 593.1890 | C_28_H_32_O_14_ | Genkwanin 4'-[O-alpha-L-rhamnopyranosyl-(1->2)-beta-D-galactopyranoside] | DS |
| 82 | 65.84 | 367.0813 | 369.0981 | C_20_H_16_O_7_ | Corylidin | BGZ |
| 83 | 66.50 | 691.3527 |  | C_32_H_54_O_13_ | Goshonoside-F5 | FPZ |
| 84 | 68.50 | 339.1228 | 341.1392 | C_20_H_20_O_5_ | Bakuchalcone | BGZ |
| 85 | 68.76 | 337.1076 | 339.1236 | C_20_H_18_O_5_ | Psoralenol/Methyl tanshinonate | BGZ/DS |
| 86 | 69.08 | 697.3637 |  | C_32_H_58_O_16_ | Cuscutic acid A2 | TSZ、NZZ |
| 87 | 69.09 | 329.2328 |  | C_18_H_34_O_5_ | Trihydroxyoctadecamonoenoic acid |  |
| 88 | 69.55 |  | 289.1806 | C_18_H_24_O_3_ | Psoracorylifol A | BGZ |
| 89 | 70.22 |  | 273.1583 | C_18_H_24_O_2_ | (+)-nimbiol | DS |
| 90 | 71.95 | 885.4326 |  | C_39_H_68_O_19_ | Reaxys ID: 7566021 | TSZ |
| 91 | 73.14 | 323.1286 | 325.1442 | C_20_H_20_O_4_ | Isobavachalcone | HQ |

DH:*Rehmannia glutinosa* Libosch.(Dihuang);NZZ: *L igustrum lucidum* Ait.; FPZ: *Rubus chingii* Hu; TSZ: *Cwscwia australis* R. Br; BGZ: *Psoralea corylifolia* L.; HQ: *Astragalus membranaceus* (Fisch.) *Bge. var. mongholicus* ( Bge. ) Hsiao; DS: *Salvia m iltiorrhiza Bge.*

* Identified with reference standard


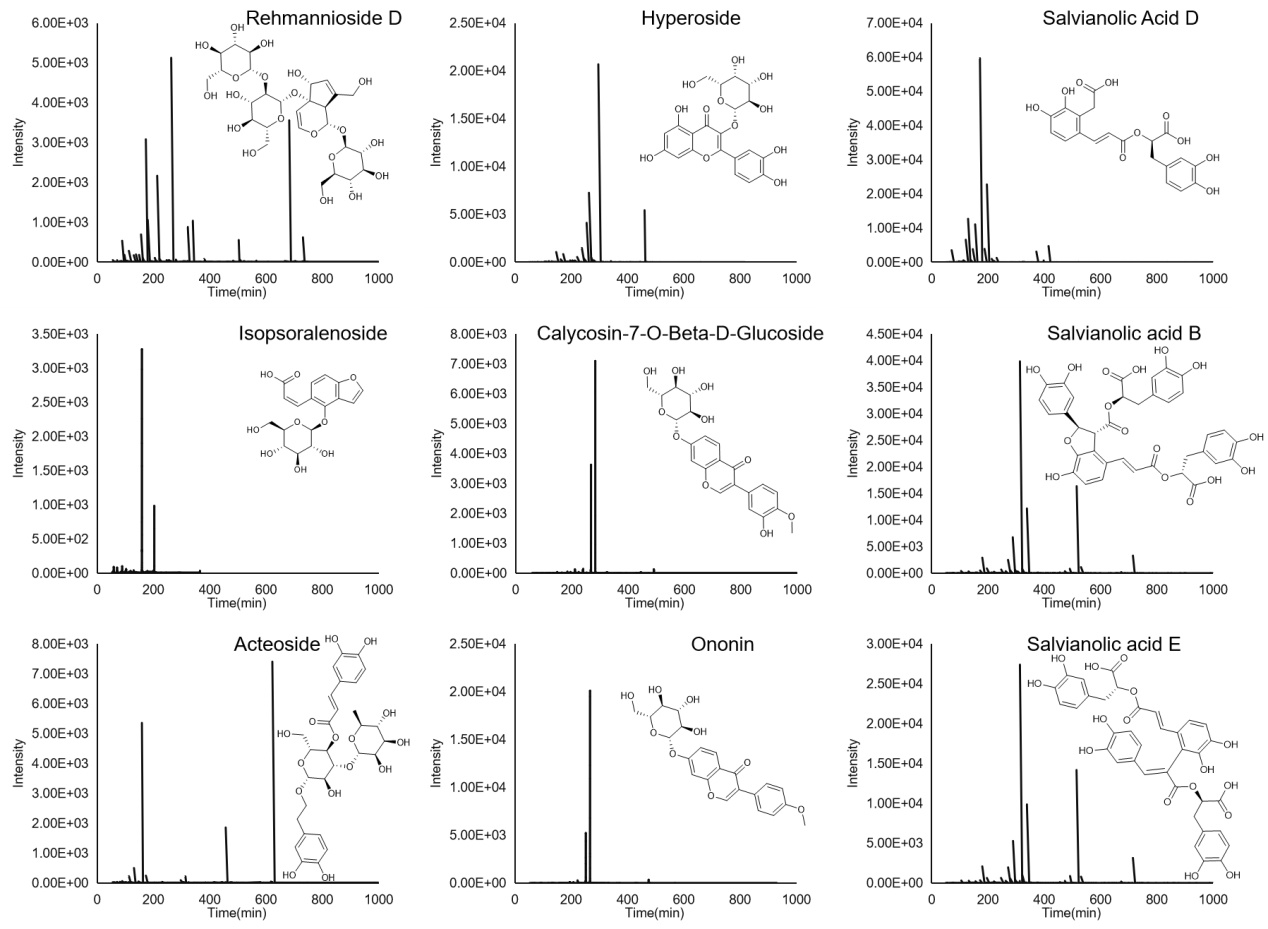


Supplementary Figure 1. Mass spectrum chromatograms of representative reference standards.
